# Behavioural, psychiatric and psychosocial factors associated with aggressive behaviour in adults with intellectual disabilities: A systematic review and narrative analysis

**DOI:** 10.1111/jar.12809

**Published:** 2020-10-18

**Authors:** Natalie van den Akker, Marieke Kroezen, Jannelien Wieland, Annelieke Pasma, Ria Wolkorte

**Affiliations:** ^1^ Intellectual Disability Medicine Department of General Practice Erasmus MC University Medical Centre Rotterdam The Netherlands; ^2^ Cordaan, Center for Excellence on Mental Health and Mild Intellectual Disability Amsterdam The Netherlands; ^3^ Department of Rheumatology Erasmus MC University Medical Centre Rotterdam The Netherlands

**Keywords:** aggression, intellectual disability, psychiatric disorders, psychiatric symptoms, psychosocial factors, self‐injurious behaviour

## Abstract

**Background:**

Aggressive behaviour is prevalent in people with intellectual disabilities. To understand the aetiology, it is important to recognize factors associated with the behaviour.

**Method:**

A systematic review was conducted and included studies published between January 2002 and April 2017 on the association of behavioural, psychiatric and psychosocial factors with aggressive behaviour in adults with intellectual disabilities.

**Results:**

Thirty‐eight studies were included that presented associations with 11 behavioural, psychiatric and psychosocial factors. Conflicting evidence was found on the association of these factors with aggressive behaviour.

**Conclusions:**

The aetiology of aggressive behaviour is specific for a certain person in a certain context and may be multifactorial. Additional research is required to identify contributing factors, to understand causal relationships and to increase knowledge on possible interaction effects of different factors.

## 1 BACKGROUND

Aggressive behaviour is common in people with intellectual disabilities (Cooper et al., 2009; Embregts et al., 2009). It is the main reason for referral to mental health services and placement in institutions (Crocker et al., 2006; Tenneij et al., 2009; Tsiouris et al., 2011). Aggressive behaviour can have serious negative consequences for people with intellectual disability, since it can impair their personal development and social relationships, which likely decreases their quality of life (Crocker et al., 2014; Embregts et al., 2009; Lundqvist, 2013). Furthermore, it often places a heavy burden on relatives and caregivers, which in turn can negatively impact the care for people with intellectual disability (Hartley & MacLean, 2007; Lundqvist, 2013).

Aggressive behaviour can manifest as different topographies, including physically aggressive behaviour, verbally aggressive behaviour, destructive behaviour, sexually aggressive behaviour and self‐injurious behaviour (Crocker et al., 2006; Sorgi et al., 1991). It is important to note that aggressive behaviour is not a disorder. It should be seen as behaviour that often serves a function for the person displaying this behaviour, although it is often not immediately clear what the cause or function of the behaviour is. To select the most effective treatment, it is imperative to understand the aetiology of the aggressive behaviour for a specific individual. This can be achieved by performing a functional assessment (Ali et al., 2014; Antonacci et al., 2008; Embregts et al., 2009; Kerr et al., 2013; Lloyd & Kennedy, 2014). A functional assessment may be descriptive or experimental in nature, but the focus of the assessment is on understanding the behaviour and all factors that may contribute to the emergence or continuation of that behaviour (Ali et al., 2014; Hanley et al., 2003; LaVigna & Willis, 2012; Lloyd & Kennedy, 2014). The results of this assessment may guide the treatment process and inform future preventive measures.

A range of factors has been suggested as contributing to the emergence or continuation of aggressive behaviour, including biological, psychological, social, developmental and environmental factors (Ali et al., 2014; Embregts et al., 2009). A better understanding of the factors that are commonly associated with aggressive behaviour in people with intellectual disability may support the functional assessment process. This review focuses on three groups of factors that have been suggested to be associated with aggressive behaviour: behavioural factors, psychiatric factors and psychosocial factors (Cooper et al., 2009; Emerson et al., 2001). These factors are all possible targets of interventions that may help to reduce or eliminate the aggressive behaviour. This review therefore aims to provide an overview of the association of behavioural, psychiatric and psychosocial factors with aggressive behaviour in adults with intellectual disability.

## 2 METHOD

### 2.1 Search strategy

This review was part of a larger research project to develop Dutch multidisciplinary guidelines concerning challenging behaviour in adults with intellectual disability. Seven databases (Embase, Medline, Web of Science, PsycINFO, Cochrane Central, CINAHL and Google Scholar [first 200 hits]) were searched for articles published between 2002 and April 2017. A wide variety of the following search terms was used: intellectual disability, challenging behaviour and different terms for behavioural, psychiatric and psychosocial factors (the detailed search strategies were developed in collaboration with a medical information specialist and can be found in Appendix 1). Search results were entered into Endnote X9 software (Clarivate Analytics) and duplicates were removed.

### 2.2 Study selection

Publications were included when the following criteria were met:

- The publication concerns people with mild to profound intellectual disability;
- The publication concerns either:
  - Methods for describing challenging behaviour or the person with intellectual disability that are not assessed by the Dutch commission of quality assessment of testing methods (COTAN); or
  - Non‐somatic factors related to the presence of challenging behaviour;
- The publication concerns adults (≥18 years) or results are presented separately for adults;
- The publication is written in Dutch, English or German.

Publications were excluded when the following criteria were met:

- The study sample consists entirely of people with a specific syndrome;
- The publication exclusively concerns an association between age, sex or degree of intellectual disability and challenging behaviour;
- The publication exclusively concerns biological factors related to the presence of challenging behaviour;
- The publication is a validity study aimed at validation within a non‐Dutch context;
- The publication is an abstract, editorial, book, dissertation, commentary or non‐systematic review.

Title and abstract of the first 100 references were screened independently by two reviewers. A sufficient level of agreement was reached (91% agreement; Cohen's *κ* = .52). Disagreements were discussed and the remaining publications were screened by a single reviewer. When in doubt, a second reviewer screened the article and disagreements were discussed until consensus was reached. All potentially relevant articles were obtained as full text and the first 20 articles were screened by two reviewers. A sufficient level of agreement was reached (90% agreement; Cohen's *κ* = .76), and the remaining articles were screened by one reviewer.

#### 2.2.1 Additional step

Only those publications included as part of the guideline development process that concerned factors related to aggressive behaviours were included in the current review. Subsequently, the reference lists of these articles were screened, with the purpose of identifying additional publications meeting the inclusion criteria for this systematic review.

### 2.3 Data synthesis and analysis

Data were extracted by two researchers. General characteristics of the study, study population, methodology, information on aggressive behaviour, information on behavioural, psychiatric and psychosocial factors and associations were extracted.

The outcome most fully adjusted for confounders was extracted. Where possible, odds ratios were reported or calculated. Otherwise, correlation or regression coefficients were presented. Where relevant, in order to correctly interpret results, the direction of association(s) was reversed.

Study type was noted as “informant report” if data were collected through questionnaires completed by or interviews held with informants, as “self‐report” if data were collected through questionnaires completed by or interviews held with people with intellectual disability themselves and as “retrospective case review” if data were collected from case files.

Data were extracted separately for five topographies of aggressive behaviour following the categories of the modified overt aggression scale (MOAS+) (Crocker et al., 2006; Sorgi et al., 1991); physically aggressive behaviour (behaviour that causes bodily harm to other people), verbally aggressive behaviour (shouting, swearing or making verbal insults), sexually aggressive behaviour (making sexually inappropriate statements, exposing oneself to others, inappropriately touching oneself or others, or engaging in coercive sexual activities), destructive behaviour (aggressive behaviour aimed at objects, or the destruction of property) or self‐injurious behaviour (behaviour that causes bodily harm to oneself). Aggressive behaviour that was not specified or specified as a combination of different topographies, was reported in the category “aggression in general.”

Behavioural factors include all reported topographies of aggressive behaviour and criminal behaviour.

Psychiatric factors were categorized as “psychiatric disorders” if the diagnosis was based on criteria outlined by the diagnostic and statistical manual (DSM) or international classification of diseases (ICD). Subcategories were created based on the ICD‐10 categorization. If a study reported an association with any psychiatric disorder, without specifying the disorder, it was classified as such. If the method of diagnosing was not specified, or when screening instruments or questionnaires were used, the results were categorized as “psychiatric symptoms.” When possible—for instance when screening instruments for a specific disorder were used—these were categorized according to the corresponding ICD‐10 categories of the respective disorders. Symptoms that were not specific to a single diagnostic category were classified as “aspecific psychiatric symptoms.” If a study reported associations with a total scale measuring symptoms of mental health problems, these were classified as “total psychiatric symptoms.”

Psychosocial factors can be described as “psychological or social variables, as well as factors pertaining to the interaction of the individual and the social environment” (Hall, 2018). These include life events, living situations, factors pertaining to social interactions and personal skills.

Considering the high heterogeneity of methodological approaches, populations, definitions, outcome measures and assessment methodologies, an overview of all associations will be provided in tables and results will be presented narratively.

### 2.4 Quality assessment

The methodological quality of the included studies was assessed using the “NIH quality assessment tool for observational cohort and cross‐sectional studies” (National Institutes of Health, 2014). After discussion of the criteria, quality assessment was performed by a single reviewer. In case of uncertainties, the second reviewer was consulted. The NIH quality assessment tool does not have a predefined cut off‐score for high or low quality. Therefore, a number of criteria have been set by the researchers. To be judged as a high‐quality study, publications had to score positively on at least seven of the 14 criteria. Furthermore, they had to score positively on three important criteria: (a) sample size justification or power calculation, (b) clearly defined, reliable and valid dependent and (c) independent variables. Tables 1, 2, 3, 4, 5, 6, 7 show the methodology quality of studies; high‐quality studies are depicted in bold, low‐quality studies are not in bold.

**TABLE 1:** Summary of included publications

Author(s) and country	Study sample	Data collection method	Type(s) of aggressive behaviour (instruments)	Psychosocial factor(s) (instruments)	Association	Outcome	Statistical analysis
Alexander et al. (2010), UK Low quality	*n* = 138 adults (109M, 29F) with mild intellectual disability and offending behaviours in an inpatient service for offenders	Retrospective chart review	Physically aggressive behaviour (case file: defined as history of aggression, recorded as either present or absent)	Psychiatric diagnosis: personality disorder (ICD‐10 diagnosis derived from case file)	NS	OR = 1.53, CI [0.49; 4.83]	Univariate, odds ratio^a^
			Verbally aggressive behaviour (case file: defined as history of aggression, recorded as either present or absent)	Psychiatric diagnosis: personality disorder (ICD‐10 diagnosis derived from case file)	NS	OR = 2.20, CI [0.50; 9.61]	Univariate, odds ratio^a^
			Destructive behaviour (case file: defined as history of aggression, recorded as either present or absent)	Psychiatric diagnosis: personality disorder (ICD‐10 diagnosis derived from case file)	NS	OR = 1.51, CI [0.52; 4.42]	Univariate, odds ratio^a^
			Self‐injurious behaviour (case file: defined as history of aggression, recorded as either present or absent)	Psychiatric diagnosis: personality disorder (ICD‐10 diagnosis derived from case file)	NS	OR = 1.47, CI [0.63; 3.41]	Univariate, odds ratio^a^
			Sexually aggressive behaviour (case file: defined as history of aggression, recorded as either present or absent)	Psychiatric diagnosis: personality disorder (ICD‐10 diagnosis derived from case file)	NS	OR = 1.79, CI [0.91; 3.54]	Univariate, odds ratio^a^
Alexander et al. (2015), UK Low quality	*n* = 138 adults (109M, 29F) with mild intellectual disability and offending behaviours in an inpatient service for offenders	Retrospective chart review	Destructive behaviour (case file: defined as history of fire setting or conviction of arson in the case history)	Life events: past experience of any abuse (evidence of child or vulnerable adult protection by Social Services)	+	OR = 2.88, CI [1.21; 6.88]	Univariate, odds ratio^a^
				Life events: past experience of sexual abuse (evidence of child or vulnerable adult protection by Social Services)	NS	OR = 1.93, CI [0.85; 4.39]	Univariate, odds ratio^a^
				Psychiatric diagnosis: PDD (ICD‐10 diagnosis derived from case file)	NS	OR = 0.50, CI [0.19; 1.34]	Univariate, odds ratio^a^
				Psychiatric diagnosis: psychosis (ICD‐10 diagnosis derived from case file)	NS	OR = 1.38, CI [0.52; 3.67]	Univariate, odds ratio^a^
				Psychiatric diagnosis: bipolar disorder (ICD‐10 diagnosis derived from case file)	NS	OR = 0.22, CI [0.03; 1.78]	Univariate, odds ratio^a^
				Psychiatric diagnosis: depressive disorder (ICD‐10 diagnosis derived from case file)	NS	OR = 1.39, CI [0.49; 3.94]	Univariate, odds ratio^a^
				Psychiatric diagnosis: substance dependence (ICD‐10 diagnosis derived from case file)	NS	OR = 1.93, CI [0.82; 4.51]	Univariate, odds ratio^a^
				Psychiatric diagnosis: personality disorder (ICD‐10 diagnosis derived from case file)	+	OR = 4.08, CI [1.54; 10.79]	Univariate, odds ratio^a^
				Criminal behaviour: history of convictions for violent offences (case file)	+	OR = 3.13, CI [1.36; 7.23]	Univariate, odds ratio^a^
				Criminal behaviour: history of convictions for destructive offences (case file)	+	OR = 185.42, CI [10.55; 3,259.22]	Univariate, odds ratio^a^
				Criminal behaviour: history of convictions for sex offences (case file)	NS	OR = 0.94, CI [0.34; 2.59]	Univariate, odds ratio^a^
				Aggressive behaviour: physical (case file: defined as a history of aggression to people, recorded as either present or absent)	NS	OR = 0.46, CI [0.13; 1.71]	Univariate, odds ratio^a^
				Aggressive behaviour: verbal (case file: defined as a history of verbal aggression, recorded as either present or absent)	NS	OR = 1.45, CI [0.16; 12.91]	Univariate, odds ratio^a^
				Aggressive behaviour: destructive (case file: defined as a history of aggression against property, recorded as either present or absent)	NS	OR = 0.41, CI [0.12; 1.37]	Univariate, odds ratio^a^
				Aggressive behaviour: sexual (case file: defined as a history of sexual aggression, recorded as either present or absent)	NS	OR = 1.90, CI [0.82; 4.38]	Univariate, odds ratio^a^
				Aggressive behaviour: self‐injurious (case file: defined as a history of aggression to self, recorded as either present or absent)	NS	OR = 2.39, CI [0.66; 8.60]	Univariate, odds ratio^a^
Allen et al. (2012), UK Low quality	*n* = 707 adults (410M, 297F) with intellectual disability and challenging behaviour (*M*_age_ = 42, range 18–93), living in different settings	Informant reports by primary carers	Destructive behaviour (Individual Schedule)	Psychiatric symptoms: affective/neurotic, possible organic (PAS‐ADD)	+	*ρ* = .081	Univariate, Spearman rank correlation
				Psychiatric symptoms: possible organic (PAS‐ADD)	+	*ρ* = .11	Univariate, Spearman rank correlation
				Psychiatric symptoms: psychotic disorder (PAS‐ADD)	NS	Not reported	Univariate, Spearman rank correlation
			Self‐injurious behaviour (Individual Schedule)	Psychiatric symptoms: affective/neurotic (PAS‐ADD)	NS	Not reported	Univariate, Spearman rank correlation
				Psychiatric symptoms: possible organic (PAS‐ADD)	NS	Not reported	Univariate, Spearman rank correlation
				Psychiatric symptoms: psychotic disorder (PAS‐ADD)	NS	Not reported	Univariate, Spearman rank correlation
			Aggressive behaviour in general (Individual Schedule)	Psychiatric symptoms: affective/neurotic (PAS‐ADD)	+	*ρ* = .10	Univariate, Spearman rank correlation
				Psychiatric symptoms: possible organic (PAS‐ADD)	+	*ρ* = .14	Univariate, Spearman rank correlation
				Psychiatric symptoms: psychotic disorder (PAS‐ADD)	NS	Not reported	Univariate, Spearman rank correlation
Bernstein et al. (2015), Hungary High quality	*n* = 50 adults (38M, 12F) with moderate, severe, or profound intellectual disability, residing in a developmental habilitation home (*M*_age_ = 31.38, *SD* = 7.63, range 19–49)	Informant reports by care staff	Physically aggressive behaviour (CBI)	Psychiatric symptoms: mood (MIPQ‐S)	NS	*ρ* = .02	Univariate, Spearman correlation
				Psychiatric symptoms: interest/pleasure (MIPQ‐S)	NS	*ρ* = −.11	Univariate, Spearman correlation
				Aggressive behaviour: general (BPI‐S)	+	*ρ* = .78	Univariate, Spearman correlation
				Aggressive behaviour: self‐injurious (BPI‐S, CBI)	NS	*ρ* = .27 (BPI‐S) *ρ* = .45 (CBI)	Univariate, Spearman correlation
			Self‐injurious behaviour (BPI‐S, CBI)	Psychiatric symptoms: mood (MIPQ‐S)	NS	*ρ* = −.17 (BPI‐S) *ρ* = −.12 (CBI)	Univariate, Spearman correlation
				Psychiatric symptoms: interest/pleasure (MIPQ‐S)	NS	*ρ* = −.44 (BPI‐S) *ρ* = −.23 (CBI)	Univariate, Spearman correlation
				Aggressive behaviour: physical (CBI)	NS	*ρ* = .45	Univariate, Spearman correlation
				Aggressive behaviour: general (BPI‐S)	+	*ρ* = .57	Univariate, Spearman correlation
			Aggressive behaviour in general (BPI‐S)	Psychiatric symptoms: mood (MIPQ‐S)	NS	*ρ* = .13	Univariate, Spearman correlation
				Psychiatric symptoms: interest/pleasure (MIPQ‐S)	NS	*ρ* = .01	Univariate, Spearman correlation
				Aggressive behaviour: physical (CBI)	+	*ρ* = .78	Univariate, Spearman correlation
				Aggressive behaviour: self‐injurious (CBI)	+	*ρ* = .57	Univariate, Spearman correlation
Bowring et al. (2017), USA Low quality	*n* = 265 adults (134M, 131F) with mild, moderate, severe, or profound intellectual disability who (had) received support from services (*M*_age_ = 41.44, *SD* = 16.28) and lived in different settings	Informant reports by family members or care staff	Self‐injurious behaviour (BPI‐S)	Communication skills: non‐verbal^b^ (Individual survey)	−	RR = 4.705, CI [1.953; 11.333]	Univariate, relative risk estimation
				Communication skills: no clear speech^b^ (Individual survey)	−	RR = 3.681, CI [1.378; 9.834]	Univariate, relative risk estimation
				Communication skills: limited understanding^b^ (Individual survey)	−	RR = 3.658, CI [1.571; 8.52]	Univariate, relative risk estimation
				Adaptive behaviour: no daytime engagement^b^ (Individual survey)	−	RR = 3.729, CI [1.48; 9.392]	Univariate, relative risk estimation
				Living situation: paid care (Individual survey)	+	RR = 3.023, CI [1.131; 8.079]	Univariate, relative risk estimation
				Living situation: with partner (Individual survey)	NS	RR = 0.301, CI [0.017; 5.202]	Univariate, relative risk estimation
				Psychiatric diagnosis: ASD (Individual survey)	NS	RR = 1.208, CI [0.454; 3.218]	Univariate, relative risk estimation
				Psychiatric diagnosis: any (Individual survey)	NS	RR = 2.256, CI [0.976; 5.212]	Univariate, relative risk estimation
				Aggressive behaviour: general (BPI‐S)	+	*ρ* = .253	Univariate, Spearman corerlation
			Aggressive behaviour in general (BPI‐S)	Aggressive behaviour: self‐injurious (BPI‐S)	+	*ρ* = .253	Univariate, Spearman corerlation
				Communication skills: limited understanding^b^ (Individual survey)	−	RR = 3.882, CI [1.761; 8.559]	Univariate, relative risk estimation
				Communication skills: non‐verbal^b^ (Individual survey)	−	RR = 3.04, CI [1.372; 6.735]	Univariate, relative risk estimation
				Communication skills: no clear speech^b^ (Individual survey)	NS	RR = 2.147, CI [0.932; 4.945]	Univariate, relative risk estimation
				Adaptive behaviour: no daytime engagement^b^ (Individual survey)	NS	RR = 1.918, CI [0.86; 4.276]	Univariate, relative risk estimation
				Living situation: paid care (Individual survey)	NS	RR = 2.159, CI [0.91; 5.124]	Univariate, relative risk estimation
				Living situation: with partner (Individual survey)	NS	RR = 0.271, CI [0.016; 4.67]	Univariate, relative risk estimation
				Psychiatric diagnosis: any (Individual survey)	NS	RR = 1.034, CI [0.421; 2.537]	Univariate, relative risk estimation
				Psychiatric diagnosis: ASD (Individual survey)	+	RR = 3.383, CI [1.544; 7.414]	Univariate, relative risk estimation
Cervantes and Matson (2015), USA High quality	*n* = 307 adults (156M, 151F) with severe or profound intellectual disability, residing in developmental centres (*M*_age_ = 51.44, *SD* = 12.49, range 20–88)	Informant reports by care staff	Sexually aggressive behaviour (DASH‐II)	Psychiatric diagnosis: ASD (DSM−5, case file)	+	*F*(1, 303) = 10.87	Multivariate, ANCOVA
			Self‐injurious behaviour (DASH‐II)	Psychiatric diagnosis: ASD (DSM‐5, case file)	+	*F*(1, 303) = 13.73	Multivariate, ANCOVA
Clark et al. (2016), Canada High quality	*n* = 215 adults with mild or moderate intellectual disability who (had) received services, living in different settings (*M*_age_ = 39.90, *SD* = 11.87, range 18–65). Participants had to be able to understand English or French	Retrospective chart review + informant reports by case managers and persons well known to participants	Aggressive behaviour in general (MOAS)	Life events: victimization history (TESI, informant reports)	+	Path coefficient = 0.99, *SE* = 0.48, *T* = 2.05	Multivariate, bootstrapped simple mediation analysis
				Psychiatric symptoms: total mental health problems (RSMB)	+	Path coefficient = 0.27, *SE* = 0.04, *T* = 6.03	Multivariate, bootstrapped simple mediation analysis
				Psychiatric symptoms: psychosis (RSMB)	+	Path coefficient = 0.86, *SE* = 0.23, *T* = 3.70	Multivariate, bootstrapped multiple mediation analysis
				Psychiatric symptoms: personality disorder (RSMB)	+	Path coefficient = 0.65, *SE* = 0.23, *T* = 2.74	Multivariate, bootstrapped multiple mediation analysis
				Psychiatric symptoms: depression (RSMB)	NS	Path coefficient = −0.37, *SE* = 0.27, *T* = −1.35	Multivariate, bootstrapped multiple mediation analysis
			Self‐injurious behaviour (MOAS)	Life events: victimization history (TESI, informant reports)	+	*t*(213) = −2.05	Univariate, *t* test
				Psychiatric symptoms: total mental health problems (RSMB)	+	Not reported	Multivariate, bootstrapped simple mediation analysis
				Psychiatric symptoms: depression (RSMB)	+	*r* = .19	Univariate, Pearson correlation
				Psychiatric symptoms: psychosis (RSMB)	+	*r* = .25	Univariate, Pearson correlation
				Psychiatric symptoms: personality disorder (RSMB)	+	*r* = .28	Univariate, Pearson correlation
Crocker et al. (2006), Canada Low quality	*n* = 3,165 adults (1,633M, 1,527F) with mild, moderate, severe, or profound intellectual disability receiving services and living in different settings (*M*_age_ = 40.63, *SD* = 13)	Informant reports by case managers and educators	Physically aggressive behaviour (MOAS)	Living situation: family (informant survey)	NS	Not reported	Univariate, *χ* ^2^‐test
				Living situation: family‐type residence (informant survey)	NS	Not reported	Univariate, *χ* ^2^‐test
				Living situation: group home (informant survey)	NS	Not reported	Univariate, *χ* ^2^‐test
				Living situation: apartment (informant survey)	NS	Not reported	Univariate, *χ* ^2^‐test
				Living situation: other (informant survey)	+	Not reported	Univariate, *χ* ^2^‐test
				Criminal behaviour: history of arrest (informant survey: rated as either present or absent)	+	Not reported	Univariate, *χ* ^2^‐test
				Aggressive behaviour: verbal (MOAS)	+	*ρ* = .53	Univariate, Spearman correlation
				Aggressive behaviour: destructive (MOAS)	+	*ρ* = .59	Univariate, Spearman correlation
				Aggressive behaviour: sexual (MOAS)	+	*ρ* = .20	Univariate, Spearman correlation
				Aggressive behaviour: self‐injurious (MOAS)	+	*ρ* = .35	Univariate, Spearman correlation
			Verbally aggressive behaviour (MOAS)	Living situation: family (informant survey)	NS	Not reported	Univariate, *χ* ^2^‐test
				Living situation: family‐type residence (informant survey)	NS	Not reported	Univariate, *χ* ^2^‐test
				Living situation: group home (informant survey)	NS	Not reported	Univariate, *χ* ^2^‐test
				Living situation: apartment (informant survey)	NS	Not reported	Univariate, *χ* ^2^‐test
				Living situation: other (informant survey)	+	Not reported	Univariate, *χ* ^2^‐test
				Criminal behaviour: history of arrest (informant survey: rated as either present or absent)	+	Not reported	Univariate, *χ* ^2^‐test
				Aggressive behaviour: physical (MOAS)	+	*ρ* = .53	Univariate, Spearman correlation
				Aggressive behaviour: destructive (MOAS)	+	*ρ* = .54	Univariate, Spearman correlation
				Aggressive behaviour: sexual (MOAS)	+	*ρ* = .21	Univariate, Spearman correlation
				Aggressive behaviour: self‐injurious (MOAS)	+	*ρ* = .26	Univariate, Spearman correlation
			Destructive behaviour (MOAS)	Living situation: family (informant survey)	NS	Not reported	Univariate, *χ* ^2^‐test
				Living situation: family‐type residence (informant survey)	NS	Not reported	Univariate, *χ* ^2^‐test
				Living situation: group home (informant survey)	NS	Not reported	Univariate, *χ* ^2^‐test
				Living situation: apartment (informant survey)	NS	Not reported	Univariate, *χ* ^2^‐test
				Living situation: other (informant survey)	+	Not reported	Univariate, *χ* ^2^‐test
				Criminal behaviour: history of arrest (informant survey: rated as either present or absent)	+	Not reported	Univariate, *χ* ^2^‐test
				Aggressive behaviour: physical (MOAS)	+	*ρ* = .59	Univariate, Spearman correlation
				Aggressive behaviour: verbal (MOAS)	+	*ρ* = .54	Univariate, Spearman correlation
				Aggressive behaviour: sexual (MOAS)	+	*ρ* = .19	Univariate, Spearman correlation
				Aggressive behaviour: self‐injurious (MOAS)	+	*ρ* = .38	Univariate, Spearman correlation
			Sexually aggressive behaviour (MOAS)	Living situation: family (informant survey)	NS	Not reported	Univariate, *χ* ^2^‐test
				Living situation: family‐type residence (informant survey)	NS	Not reported	Univariate, *χ* ^2^‐test
				Living situation: group home (informant survey)	NS	Not reported	Univariate, *χ* ^2^‐test
				Living situation: apartment (informant survey)	NS	Not reported	Univariate, *χ* ^2^‐test
				Living situation: other (informant survey)	+	Not reported	Univariate, *χ* ^2^‐test
				Criminal behaviour: history of arrest (informant survey: rated as either present or absent)	+	Not reported	Univariate, *χ* ^2^‐test
				Aggressive behaviour: physical (MOAS)	+	*ρ* = .20	Univariate, Spearman correlation
				Aggressive behaviour: verbal (MOAS)	+	*ρ* = .21	Univariate, Spearman correlation
				Aggressive behaviour: destructive (MOAS)	+	*ρ* = .19	Univariate, Spearman correlation
				Aggressive behaviour: self‐injurious (MOAS)	+	*ρ* = .13	Univariate, Spearman correlation
			Self‐injurious behaviour (MOAS)	Living situation: family (informant survey)	NS	Not reported	Univariate, *χ* ^2^‐test
				Living situation: family‐type residence (informant survey)	NS	Not reported	Univariate, *χ* ^2^‐test
				Living situation: group home (informant survey)	NS	Not reported	Univariate, *χ* ^2^‐test
				Living situation: apartment (informant survey)	NS	Not reported	Univariate, *χ* ^2^‐test
				Living situation: other (informant survey)	+	Not reported	Univariate, *χ* ^2^‐test
				Criminal behaviour: history of arrest (informant survey: rated as either present or absent)	NS	Not reported	Univariate, *χ* ^2^‐test
				Aggressive behaviour: physical (MOAS)	+	*ρ* = .35	Univariate, Spearman correlation
				Aggressive behaviour: verbal (MOAS)	+	*ρ* = .26	Univariate, Spearman correlation
				Aggressive behaviour: destructive (MOAS)	+	*ρ* = .38	Univariate, Spearman correlation
				Aggressive behaviour: sexual (MOAS)	+	*ρ* = .13	Univariate, Spearman correlation
			Aggressive behaviour in general (MOAS)	Living situation: family (informant survey)	NS	Not reported	Univariate, *χ* ^2^‐test
				Living situation: family‐type residence (informant survey)	NS	Not reported	Univariate, *χ* ^2^‐test
				Living situation: group home (informant survey)	NS	Not reported	Univariate, *χ* ^2^‐test
				Living situation: apartment (informant survey)	NS	Not reported	Univariate, *χ* ^2^‐test
				Living situation: other (informant survey)	+	Not reported	Univariate, *χ* ^2^‐test
				Criminal behaviour: history of arrest (informant survey: rated as either present or absent)	+	*t*(137.91) = −5.84	Univariate, *t* test
Crocker et al. (2014), Canada High quality	*n* = 296 adults (162M, 134F) with mild or moderate intellectual disability living in the community and receiving services (*M*_age_ = 40.67, *SD* = 12.21, range 18–65). Participants had to be able to understand English or French	Retrospective chart review + self‐reports + informant reports by a case manager and significant others	Physically aggressive behaviour (MOAS)	Psychiatric diagnosis: number of mental disorders (case file)	NS	Incidence rate ratio = 1.450, CI [0.980; 2.146]	Multivariate, logistic regression
				Psychiatric diagnosis: severity of mental disorders (SF‐36)	NS	Incidence rate ratio = 0.972, CI [0.936; 1.009]	Multivariate, logistic regression
			Verbally aggressive behaviour (MOAS)	Psychiatric diagnosis: number of mental disorders (case file)	+	Incidence rate ratio = 3.200, CI [1.294; 7.914]	Multivariate, logistic regression
				Psychiatric diagnosis: severity of mental disorders (SF‐36)	+	Incidence rate ratio = 0.937, CI [0.890; 0.986]	Multivariate, logistic regression
			Destructive behaviour (MOAS)	Psychiatric diagnosis: number of mental disorders (case file)	NS	Incidence rate ratio = 1.258, CI [0.849; 1.863]	Multivariate, logistic regression
				Psychiatric diagnosis: severity of mental disorders (SF‐36)	−	Incidence rate ratio = 0.956, CI [0.920; 0.993]	Multivariate, logistic regression
			Sexually aggressive behaviour (MOAS)	Psychiatric diagnosis: anxiety disorder (SF‐36, case file)	+	Incidence rate ratio = 3.224, CI [1.311; 7.923]	Multivariate, logistic regression
Davies et al. (2015), UK High quality	*n* = 96 adults (50M, 46F) with mild or moderate intellectual disability (*M*_age_ = 39.68, *SD* = 13.32, range 18–79). Participants had to be able to complete the questionnaires	Self‐reports + informant reports by carers	Aggressive behaviour in general (CCB)	Psychiatric symptoms: alexithymia (self‐report using AQC)	NS	*ρ* = .133	Univariate, Spearman correlation
				Psychiatric symptoms: alexithymia (informant report using OAS)	+	*ρ* = .298	Univariate, Spearman correlation
Didden et al. (2009), the Netherlands Low quality	*n* = 39 adult inpatients of a specialized treatment unit, with mild intellectual disability (age range 19–51)	Retrospective chart review	Aggressive behaviour in general (ABCL)	Psychiatric symptoms: substance abuse (case file: use of much more than 14 (females) or 21 (males) standard units of alcohol per week, with similar criteria for drug use)	+	*z* = 2.187	Univariate, Mann–Whitney analysis
Drieschner et al. (2013), the Netherlands Low quality	*n* = 218 adults (188M, 30F) with mild intellectual disability, living in residential treatment centres for adults with intellectual disability who display serious dangerous behaviour (*M*_age_ = 33.8, *SD* = 11.5)	Informant reports	Physically aggressive behaviour (MOAS+)	Aggressive behaviour: verbal (MOAS+)	+	*ρ* = .70	Univariate, Spearman correlation
				Aggressive behaviour: destructive (MOAS+)	+	*ρ* = .73	Univariate, Spearman correlation
				Aggressive behaviour: sexual (MOAS+)	+	*ρ* = .30	Univariate, Spearman correlation
				Aggressive behaviour: self‐injurious (MOAS+)	+	*ρ* = .47	Univariate, Spearman correlation
				Psychiatric diagnosis: ADHD (DSM‐IV)	+	Incidence rate ratio = 2.53	Univariate, Mann–Whitney *U*‐test
				Psychiatric diagnosis: Borderline personality disorder (DSM‐IV)	NS	Not reported	Univariate, Mann–Whitney *U*‐test
				Psychiatric diagnosis: substance‐related disorder (DSM‐IV)	NS	Not reported	Univariate, Mann–Whitney *U*‐test
				Psychiatric diagnosis: psychotic disorder (DSM‐IV)	NS	Not reported	Univariate, Mann–Whitney *U*‐test
				Psychiatric diagnosis: mood or anxiety disorder (DSM‐IV)	NS	Not reported	Univariate, Mann–Whitney *U*‐test
				Psychiatric diagnosis: PDD (DSM‐IV)	NS	Not reported	Univariate, Mann–Whitney *U*‐test
				Psychiatric diagnosis: paraphilia (DSM‐IV)	NS	Not reported	Univariate, Mann–Whitney *U*‐test
				Psychiatric diagnosis: antisocial personality disorder (DSM‐IV)	NS	Not reported	Univariate, Mann–Whitney *U*‐test
				Criminal behaviour: admission on the basis of criminal law (informant reports)	‐	Incidence rate ratio = −1.86	Univariate, Mann–Whitney *U*‐test
			Verbally aggressive behaviour (MOAS+)	Aggressive behaviour: physical (MOAS+)	+	*ρ* = .70	Univariate, Spearman correlation
				Aggressive behaviour: destructive (MOAS+)	+	*ρ* = .80	Univariate, Spearman correlation
				Aggressive behaviour: sexual (MOAS+)	+	*ρ* = .35	Univariate, Spearman correlation
				Aggressive behaviour: self‐injurious (MOAS+)	+	*ρ* = .39	Univariate, Spearman correlation
				Psychiatric diagnosis: ADHD (DSM‐IV)	+	Incidence rate ratio = 1.88	Univariate, Mann–Whitney *U*‐test
				Psychiatric diagnosis: Borderline personality disorder (DSM‐IV)	NS	Not reported	Univariate, Mann–Whitney *U*‐test
				Psychiatric diagnosis: substance‐related disorder (DSM‐IV)	NS	Not reported	Univariate, Mann–Whitney *U*‐test
				Psychiatric diagnosis: psychotic disorder (DSM‐IV)	NS	Not reported	Univariate, Mann–Whitney *U*‐test
				Psychiatric diagnosis: mood or anxiety disorder (DSM‐IV)	NS	Not reported	Univariate, Mann–Whitney *U*‐test
				Psychiatric diagnosis: PDD (DSM‐IV)	NS	Not reported	Univariate, Mann–Whitney *U*‐test
				Psychiatric diagnosis: paraphilia (DSM‐IV)	NS	Not reported	Univariate, Mann–Whitney *U*‐test
				Psychiatric diagnosis: antisocial personality disorder (DSM‐IV)	NS	Not reported	Univariate, Mann–Whitney *U*‐test
				Criminal behaviour: admission on the basis of criminal law (informant reports)	−	Incidence rate ratio = −1.59	Univariate, Mann–Whitney *U*‐test
			Destructive behaviour (MOAS+)	Aggressive behaviour: physical (MOAS+)	+	*ρ* = .73	Univariate, Spearman correlation
				Aggressive behaviour: verbal (MOAS+)	+	*ρ* = .80	Univariate, Spearman correlation
				Aggressive behaviour: sexual (MOAS+)	+	*ρ* = .29	Univariate, Spearman correlation
				Aggressive behaviour: self‐injurious (MOAS+)	+	*ρ* = .50	Univariate, Spearman correlation
				Psychiatric diagnosis: ADHD (DSM‐IV)	+	Incidence rate ratio = 2.75	Univariate, Mann–Whitney *U*‐test
				Psychiatric diagnosis: Borderline personality disorder (DSM‐IV)	NS	Not reported	Univariate, Mann–Whitney *U*‐test
				Psychiatric diagnosis: substance‐related disorder (DSM‐IV)	−	Incidence rate ratio = −1.67	Univariate, Mann–Whitney *U*‐test
				Psychiatric diagnosis: psychotic disorder (DSM‐IV)	NS	Not reported	Univariate, Mann–Whitney *U*‐test
				Psychiatric diagnosis: mood or anxiety disorder (DSM‐IV)	NS	Not reported	Univariate, Mann–Whitney *U*‐test
				Psychiatric diagnosis: PDD (DSM‐IV)	NS	Not reported	Univariate, Mann–Whitney *U*‐test
				Psychiatric diagnosis: paraphilia (DSM‐IV)	NS	Not reported	Univariate, Mann–Whitney *U*‐test
				Psychiatric diagnosis: antisocial personality disorder (DSM‐IV)	NS	Not reported	Univariate, Mann–Whitney *U*‐test
				Criminal behaviour: admission on the basis of criminal law (informant reports)	−	Incidence rate ratio = −2.06	Univariate, Mann–Whitney *U*‐test
			Sexually aggressive behaviour (MOAS+)	Aggressive behaviour: physical (MOAS+)	+	*ρ* = .30	Univariate, Spearman correlation
				Aggressive behaviour: verbal (MOAS+)	+	*ρ* = .35	Univariate, Spearman correlation
				Aggressive behaviour: destructive (MOAS+)	+	*ρ* = .29	Univariate, Spearman correlation
				Aggressive behaviour: self‐injurious (MOAS+)	+	*ρ* = .24	Univariate, Spearman correlation
				Psychiatric diagnosis: ADHD (DSM‐IV)	+	Incidence rate ratio = 3.08	Univariate, Mann–Whitney *U*‐test
				Psychiatric diagnosis: Borderline personality disorder (DSM‐IV)	NS	Not reported	Univariate, Mann–Whitney *U*‐test
				Psychiatric diagnosis: substance‐related disorder (DSM‐IV)	−	Incidence rate ratio = −1.45	Univariate, Mann–Whitney *U*‐test
				Psychiatric diagnosis: psychotic disorder (DSM‐IV)	NS	Not reported	Univariate, Mann–Whitney *U*‐test
				Psychiatric diagnosis: mood or anxiety disorder (DSM‐IV)	NS	Not reported	Univariate, Mann–Whitney *U*‐test
				Psychiatric diagnosis: PDD (DSM‐IV)	NS	Not reported	Univariate, Mann–Whitney *U*‐test
				Psychiatric diagnosis: paraphilia (DSM‐IV)	NS	Not reported	Univariate, Mann–Whitney *U*‐test
				Psychiatric diagnosis: antisocial personality disorder (DSM‐IV)	NS	Not reported	Univariate, Mann–Whitney *U*‐test
				Criminal behaviour: admission on the basis of criminal law (informant reports)	NS	Not reported	Univariate, Mann–Whitney *U*‐test
			Self‐injurious behaviour (MOAS+)	Aggressive behaviour: physical (MOAS+)	+	*ρ* = .47	Univariate, Spearman correlation
				Aggressive behaviour: verbal (MOAS+)	+	*ρ* = .39	Univariate, Spearman correlation
				Aggressive behaviour: destructive (MOAS+)	+	*ρ* = .50	Univariate, Spearman correlation
				Aggressive behaviour: sexual (MOAS+)	+	*ρ* = .24	Univariate, Spearman correlation
				Psychiatric diagnosis: ADHD (DSM‐IV)	+	Incidence rate ratio = 5.71	Univariate, Mann–Whitney *U*‐test
				Psychiatric diagnosis: borderline personality disorder (DSM‐IV)	+	Incidence rate ratio = 4.29	Univariate, Mann–Whitney *U*‐test
				Psychiatric diagnosis: substance‐related disorder (DSM‐IV)	NS	Not reported	Univariate, Mann–Whitney *U*‐test
				Psychiatric diagnosis: psychotic disorder (DSM‐IV)	NS	Not reported	Univariate, Mann–Whitney *U*‐test
				Psychiatric diagnosis: mood or anxiety disorder (DSM‐IV)	NS	Not reported	Univariate, Mann–Whitney *U*‐test
				Psychiatric diagnosis: PDD (DSM‐IV)	NS	Not reported	Univariate, Mann–Whitney *U*‐test
				Psychiatric diagnosis: paraphilia (DSM‐IV)	NS	Not reported	Univariate, Mann–Whitney *U*‐test
				Psychiatric diagnosis: antisocial personality disorder (DSM‐IV)	NS	Not reported	Univariate, Mann–Whitney *U*‐test
				Criminal behaviour: admission on the basis of criminal law (informant reports)	−	Incidence rate ratio = −2.85	Univariate, Mann–Whitney *U*‐test
			Aggressive behaviour in general (MOAS+)	Psychiatric diagnosis: ADHD (DSM‐IV)	+	Incidence rate ratio = 2.28	Univariate, Mann–Whitney *U*‐test
				Psychiatric diagnosis: Borderline personality disorder (DSM‐IV)	NS	Not reported	Univariate, Mann–Whitney *U*‐test
				Psychiatric diagnosis: substance‐related disorder (DSM‐IV)	−	Incidence rate ratio = −1.57	Univariate, Mann–Whitney *U*‐test
				Psychiatric diagnosis: psychotic disorder (DSM‐IV)	NS	Not reported	Univariate, Mann–Whitney *U*‐test
				Psychiatric diagnosis: mood or anxiety disorder (DSM‐IV)	NS	Not reported	Univariate, Mann–Whitney *U*‐test
				Psychiatric diagnosis: PDD (DSM‐IV)	NS	Not reported	Univariate, Mann–Whitney *U*‐test
				Psychiatric diagnosis: paraphilia (DSM‐IV)	NS	Not reported	Univariate, Mann–Whitney *U*‐test
				Psychiatric diagnosis: antisocial personality disorder (DSM‐IV)	NS	Not reported	Univariate, Mann–Whitney *U*‐test
				Criminal behaviour: admission on the basis of criminal law (informant reports)	−	Incidence rate ratio = −1.70	Univariate, Mann–Whitney *U*‐test
Esbensen and Benson (2006), USA High quality	*n* = 104 adults (58M, 46F) with mild, moderate, or severe intellectual disability (*M*_age_ = 42.0, *SD* = 12.4, range 21–79 years) and living in different settings	Informant reports by care staff	Aggressive behaviour in general (SIB‐R externalized)	Life events: positive life events (LES)	NS	*r* = .05	Univariate, Pearson correlation
				Life events: negative life events (LES)	+	*r* = .39	Univariate, Pearson correlation
				Life events: total life events (LES)	+	*r* = .24	Univariate, Pearson correlation
Hartley and MacLean (2007), USA High quality	*n* = 132 adults ≥50 years (66M, 66F, *M*_age_ = 59.22, *SD* = 7.60), with mild, moderate, severe, or profound intellectual disability receiving services and living in different settings	Informant reports by care staff	Physically aggressive behaviour (ICAP)	Adaptive behaviour: motor skills, social and communication skills, personal living skills, community living skills (ICAP Broad Independence age equivalent)	−	*τ* = −.32	Univariate, Kendall Tau C correlation
			Destructive behaviour (ICAP)	Adaptive behaviour: motor skills, social and communication skills, personal living skills, community living skills (ICAP Broad Independence age equivalent)	−	*τ* = −.29	Univariate, Kendall Tau C correlation
Hemmings et al. (2006), UK High quality	*n* = 214 adults (108M, 106F) with mild/moderate or severe/profound intellectual disability (range 18–85 years), living in a variety of settings	Retrospective chart review + self‐reports	Destructive behaviour (DAS)	Psychiatric symptoms: low energy (PAS‐ADD Checklist)	+	OR = 4.36, CI [1.43; 13.3]	Multivariate, stepwise logistic regression
				Psychiatric symptoms: delayed sleep (PAS‐ADD Checklist)	+	OR = 3.28, CI [1.1; 9.76]	Multivariate, stepwise logistic regression
				Psychiatric symptoms: anhedonia, sad or down, fearful/panicky, repetitive actions, too high or happy, suicidal, loss of appetite, weight change, loss of confidence, avoiding social contact, worthlessness, early waking, restlessness, irritable mood, loss of self‐care, odd language (PAS‐ADD Checklist)	NS	Not reported	Multivariate, stepwise logistic regression
				Social skills: social functioning (DAS)	−	OR = 4.09, CI [1.7; 9.82]	Multivariate, stepwise logistic regression
			Self‐injurious behaviour (DAS)	Psychiatric symptoms: irritable mood (PAS‐ADD Checklist)	+	OR = 5.52, CI [1.99; 15.3]	Multivariate, stepwise logistic regression
				Psychiatric symptoms: suicidal (PAS‐ADD Checklist)	+	OR = 5.19, CI [1.22; 22.1]	Multivariate, stepwise logistic regression
				Psychiatric symptoms: low energy, anhedonia, sad or down, fearful/panicky, repetitive actions, too high or happy, loss of appetite, weight change, loss of confidence, avoiding social contact, worthlessness, delayed sleep, early waking, restlessness, loss of self‐care, odd language (PAS‐ADD Checklist)	NS	Not reported	Multivariate, stepwise logistic regression
				Social skills: social functioning (DAS)	NS	Not reported	Multivariate, stepwise logistic regression
			Aggressive behaviour in general (DAS)	Psychiatric symptoms: early waking (PAS‐ADD Checklist)	+	OR = 4.04, CI [1.08; 15.1]	Multivariate, stepwise logistic regression
				Psychiatric symptoms: low energy (PAS‐ADD Checklist)	+	OR = 3.72, CI [1.21; 11.4]	Multivariate, stepwise logistic regression
				Psychiatric symptoms: irritable mood (PAS‐ADD Checklist)	+	OR = 3.0, CI [1.16; 7.8]	Multivariate, stepwise logistic regression
				Psychiatric symptoms: anhedonia, sad or down, fearful/panicky, repetitive actions, too high or happy, suicidal, loss of appetite, weight change, loss of confidence, avoiding social contact, worthlessness, delayed sleep, restlessness, loss of self‐care, odd language (PAS‐ADD Checklist)	NS	Not reported	Multivariate, stepwise logistic regression
				Social skills: social functioning (DAS)	NS	Not reported	Multivariate, stepwise logistic regression
Horovitz et al. (2013), USA High quality	*n* = 175 adults (94M, 81F) with mild, moderate, severe, or profound intellectual disability residing in developmental centres (*M*_age_ = 52.18, *SD* = 13.41, range 20–87 years)	Informant reports by care staff	Self‐injurious behaviour (ASD‐BPA)	Psychiatric diagnosis: ASD (DSM‐IV‐TR and ICD‐10)	+	*F*(1, 170) = 11.28	Multivariate, two‐way between‐subjects ANOVA
			Aggressive behaviour in general (ASD‐BPA)	Psychiatric diagnosis: ASD (DSM‐IV‐TR and ICD‐10)	NS	*F*(1, 170) = 2.11	Multivariate, two‐way between‐subjects ANOVA
Hurley (2008), USA Low quality	*n* = 300 patients with mild, moderate, severe, or profound intellectual disability seen in a specialty clinic of a medical centre	Retrospective chart review	Self‐injurious behaviour (case file: any form of self‐injurious behaviour, excluding suicidality but including skin picking)	Psychiatric diagnosis: depression (DSM‐IV, DSM‐IV‐TR diagnosis derived from case file)	+	OR = 8.53, CI [1.09; 66.75]	Univariate, odds ratio^a^
			Aggressive behaviour in general (case file: any physical aggression towards others, objects, or verbal threats of aggression)	Psychiatric diagnosis: depression (DSM‐IV, DSM‐IV‐TR diagnosis derived from case file)	+	OR = 21.02, CI [2.73; 162.09]	Univariate, odds ratio^a^
Koritsas and Iacono (2015), Australia High quality	*n* = 74 adults (49M, 25F) with intellectual disability (*M*_age_ = 36.56, *SD* = 13.14, range 19–73 years) and living in different settings	Informant reports by care staff + brief observation	Aggressive behaviour in general (Interview Protocol, ICAP, CCB)	Psychiatric symptoms: anxiety (DBC‐A)	+	*β* = 0.52, *SE* = 0.06, *t* = 4.16	Multivariate, multiple regression
				Psychiatric symptoms: disruption (DBC‐A)	+	*ρ* = .28	Univariate, Spearman correlation
				Psychiatric symptoms: total (DBC‐A)	+	*ρ* = .24	Univariate, Spearman correlation
				Psychiatric symptoms: depressive (DBC‐A)	NS	*β* = −0.16, *SE* = 0.03, *t* = −1.36	Multivariate, multiple regression
				Psychiatric symptoms: self‐absorbed (DBC‐A)	NS	*ρ* = .19	Univariate, Spearman correlation
				Psychiatric symptoms: communication disturbance (DBC‐A)	NS	*ρ* = .12	Univariate, Spearman correlation
				Psychiatric symptoms: social relating (DBC‐A)	NS	*ρ* = .02	Univariate, Spearman correlation
				Communication skills: ability to make needs known (informant report about communication forms and functions, combined with brief observations. Overall judgment of communication skills was determined by a speech pathologist based on these instruments)	NS	*ρ* = .06	Univariate, Spearman correlation
				Living situation: with parents (compared to not living with parents) (questionnaire)	NS	*ρ* = .14	Univariate, Spearman correlation
				Aggressive behaviour: learned function of aggressive behaviour (sensory) (MAS)	NS	*β* = −0.22, *SE* = 0.02, *t* = −1.78	Multivariate, multiple regression
				Aggressive behaviour: learned function of aggressive behaviour (escape) (MAS)	NS	*β* = −0.06, *SE* = 0.03, *t* = 0.41	Multivariate, multiple regression
				Aggressive behaviour: learned function of aggressive behaviour (attention) (MAS)	NS	*β* = 0.14, *SE* = 0.03, *t* = −0.32	Multivariate, multiple regression
				Aggressive behaviour: learned function of aggressive behaviour (tangible) (MAS)	NS	*ρ* = .18	Univariate, Spearman correlation
Larson et al. (2011), UK Low quality	*n* = 60 adults (31M, 29F) with mild or moderate intellectual disability, that had to be able to read and respond to the questionnaire independently; *n* = 39 supporting persons	Informant reports by supporting persons + self‐reports	Aggressive behaviour in general (questionnaire: not specified, challenging behaviour selected from a list of commonly occurring examples of challenging behaviour)	Psychiatric symptoms: attachment style (questionnaire: secure, insecure‐anxious/ambivalent, or insecure‐avoidant)	NS	Not reported	Univariate, *χ* ^2^‐test
			Self‐injurious behaviour (questionnaire: behaviour not specified, challenging behaviour selected from a list of commonly occurring examples of challenging behaviour)	Psychiatric symptoms: attachment style (questionnaire: secure, insecure‐anxious/ambivalent, or insecure‐avoidant)	NS	Not reported	Univariate, *χ* ^2^‐test
Lindsay et al. (2013), UK Low quality	*n* = 477 adults referred to maximum secure services for antisocial or offending behaviour	Retrospective chart review	Physically aggressive behaviour (case file: behaviour leading to referral to maximum secure services	Psychiatric diagnosis: ADHD (case file)	+	OR = 1.76, CI [1.06; 2.93]	Univariate, odds ratio^a^
			Verbally aggressive behaviour (case file: behaviour leading to referral to maximum secure services)	Psychiatric diagnosis: ADHD (case file)	NS	OR = 0.85, CI [0.49; 1.46]	Univariate, odds ratio^a^
			Destructive behaviour (case file: behaviour leading to referral to maximum secure services)	Psychiatric diagnosis: ADHD (case file)	+	OR = 1.77, CI [1.00; 3.14]	Univariate, odds ratio^a^
			Sexually aggressive behaviour (case file: behaviour leading to referral to maximum secure services)	Psychiatric diagnosis: ADHD (case file)	NS	*Contact sex* OR = 0.81, CI [0.38; 1.71] *Non‐contact sex* OR = 0.72, CI [0.33; 1.58]	Univariate, odds ratio^a^
Lundqvist (2013), Sweden Low quality	*n* = 915 adults (504M, 411F) with mild, moderate, or severe/profound intellectual disability receiving care from local health authorities and living in different settings (*M*_age_ = 43.4, *SD* = 14.8, range 18–87 years)	Informant reports by care staff	Self‐injurious behaviour (BPI)	Psychiatric symptoms: autism (questionnaire based on the ICF)	+	OR = 1.70, CI [1.03; 2.80]	Multivariate, backward stepwise likelihood ratio multiple logistic regression
				Psychiatric symptoms: schizophrenia (questionnaire based on the ICF)	NS	OR = 1.61, CI [0.51; 5.13]	Univariate, binary logistic regression
				Psychiatric symptoms: psychosis (questionnaire based on the ICF)	NS	OR = 0.00, CI not reported	Univariate, binary logistic regression
				Psychiatric symptoms: depression (questionnaire based on the ICF)	NS	OR = 0.28, CI [0.03; 2.22]	Univariate, binary logistic regression
				Psychiatric symptoms: OCD (questionnaire based on the ICF)	NS	OR = 0.64, CI [0.13; 3.08]	Univariate, binary logistic regression
				Psychiatric symptoms: ADHD (questionnaire based on the ICF)	NS	Not reported	Multivariate, backward stepwise likelihood ratio multiple logistic regression
				Psychiatric symptoms: general psychopathology (questionnaire based on the ICF)	NS	Not reported	Multivariate, backward stepwise likelihood ratio multiple logistic regression
				Communication skills: communicating in writing (questionnaire based on the ICF)	NS	Not reported	Multivariate, backward stepwise likelihood ratio multiple logistic regression
				Communication skills: communicating with speech (questionnaire based on the ICF)	NS	Not reported	Multivariate, backward stepwise likelihood ratio multiple logistic regression
				Communication skills: communicating with signs (questionnaire based on the ICF)	NS	Not reported	Multivariate, backward stepwise likelihood ratio multiple logistic regression
				Communication skills: communicating with gestures (questionnaire based on the ICF)	NS	Not reported	Multivariate, backward stepwise likelihood ratio multiple logistic regression
				Communication skills: communicating with sounds (questionnaire based on the ICF)	NS	Not reported	Multivariate, backward stepwise likelihood ratio multiple logistic regression
				Communication skills: communicating with pictures (questionnaire based on the ICF)	+	OR = 1.93, CI [1.21; 3.09]	Multivariate, backward stepwise likelihood ratio multiple logistic regression
				Social skills: group functioning (questionnaire based on the ICF)	NS	Not reported	Multivariate, backward stepwise likelihood ratio multiple logistic regression
				Social skills: initiating social interaction (questionnaire based on the ICF, rated on a five‐point scale from never to always)	NS	Not reported	Multivariate, backward stepwise likelihood ratio multiple logistic regression
			Aggressive behaviour in general (BPI)	Psychiatric symptoms: autism (questionnaire based on the ICF)	+	OR = 1.78, CI [1.14; 2.77]	Multivariate, backward stepwise likelihood ratio multiple logistic regression
				Psychiatric symptoms: schizophrenia (questionnaire based on the ICF)	NS	OR = 1.92, CI [0.62; 6.01]	Univariate, binary logistic regression
				Psychiatric symptoms: psychosis (questionnaire based on the ICF)	NS	OR = 2.40, CI [0.64; 9.01]	Univariate, binary logistic regression
				Psychiatric symptoms: depression (questionnaire based on the ICF)	NS	OR = 2.40, CI [0.64; 9.01]	Univariate, binary logistic regression
				Psychiatric symptoms: OCD (questionnaire based on the ICF)	NS	OR = 0.96, CI [0.24; 3.85]	Univariate, binary logistic regression
				Psychiatric symptoms: ADHD (questionnaire based on the ICF)	NS	OR = 1.15, CI [0.55; 2.38]	Univariate, binary logistic regression
				Psychiatric symptoms: general psychopathology (questionnaire based on the ICF)	NS	Not reported	Multivariate, backward stepwise likelihood ratio multiple logistic regression
				Communication skills: communicating in writing (questionnaire based on the ICF)	NS	OR = 1.12, CI [0.79; 1.58]	Univariate, binary logistic regression
				Communication skills: communicating with speech (questionnaire based on the ICF)	NS	Not reported	Multivariate, backward stepwise likelihood ratio multiple logistic regression
				Communication skills: communicating with signs (questionnaire based on the ICF)	+	OR = 2.28, CI [1.49; 3.49]	Multivariate, backward stepwise likelihood ratio multiple logistic regression
				Communication skills: communicating with gestures (questionnaire based on the ICF)	NS	Not reported	Multivariate, backward stepwise likelihood ratio multiple logistic regression
				Communication skills: communicating with sounds (questionnaire based on the ICF)	NS	Not reported	Multivariate, backward stepwise likelihood ratio multiple logistic regression
				Communication skills: communicating with pictures (questionnaire based on the ICF)	NS	Not reported	Multivariate, backward stepwise likelihood ratio multiple logistic regression
				Social skills: group functioning (questionnaire based on the ICF)	−	OR = 0.54, CI [0.46; 0.64]	Multivariate, backward stepwise likelihood ratio multiple logistic regression
				Social skills: initiating social interaction (questionnaire based on the ICF, rated on a five‐point scale from never to always)	+	OR = 1.27, CI [1.10; 1.48]	Multivariate, backward stepwise likelihood ratio multiple logistic regression
Lunsky et al. (2012), Canada Low quality	*n* = 747 adults with mild or moderate/severe intellectual disability that have experienced crisis and living in different settings	Retrospective chart review + informant reports by care staff	Physically aggressive behaviour (case file, informant report: written description of what led up to the crisis, the crisis itself and the outcome of the crisis)	Criminal behaviour: history of legal involvement (case file)	NS	*b* = −0.247, OR = 0.781, CI [0.477; 1.280]	Multivariate, logistic regressions
				Psychiatric diagnosis: autism (case file)	NS	*b* = −0.329, OR = 0.720, CI [0.479; 1.081]	Multivariate, logistic regressions
				Psychiatric diagnosis: substance abuse disorder (case file)	NS	*b* = −0.714, OR = 0.490, CI [0.124; 1.930]	Multivariate, logistic regressions
				Living situation: minimal support (compared to group home) (case file)	−	*b* = −0.617, OR = 0.540, CI [0.337; 0.864]	Multivariate, logistic regressions
				Living situation: with family (compared to group home) (case file)	NS	*b* = −0.245, OR = 0.783, CI [0.496; 1.235]	Multivariate, logistic regressions
				Life events: negative life events (modified PAS‐ADD Checklist)	NS	*One life event* *b* = 0.010, OR = 1.010, CI [0.645; 1.583] *Two or more life events* *b* = 0.098, OR = 1.103, CI [0.719; 1.693]	Multivariate, logistic regressions
			Destructive behaviour (case file, informant report: written description of what led up to the crisis, the crisis itself and the outcome of the crisis)	Criminal behaviour: history of legal involvement (case file)	+	*χ* ^2^(1) = 6.428	Univariate, *χ* ^2^‐test
			Self‐injurious behaviour (case file, informant report: written description of what led up to the crisis, the crisis itself and the outcome of the crisis)	Criminal behaviour: history of legal involvement (case file)	+	*χ* ^2^(1) = 5.966	Univariate, *χ* ^2^‐test
Matson and Rivet (2008), USA High quality	*n* = 298 adults (167M, 131F) with mild, moderate, severe, or profound intellectual disability residing in a developmental centre (*M*_age_ = 52.03, *SD* = 12.78, range 21–88 years)	Informant reports by care staff	Self‐injurious behaviour (ASD‐BPA)	Psychiatric symptoms: restricted/repetitive behaviour (ASD‐DA)	+	*B* = 0.11, *SE* = 0.03, *β* = 0.32	Multivariate, multiple regression
				Psychiatric symptoms: social impairment (ASD‐DA)	NS	*B* = 0.02, *SE* = 0.02, *β* = 0.10	Multivariate, multiple regression
				Psychiatric symptoms: communication impairment (ASD‐DA)	NS	*B* = −0.03, *SE* = 0.03, *β* = −0.09	Multivariate, multiple regression
			Aggressive behaviour in general (ASD‐BPA)	Psychiatric symptoms: communication impairment (ASD‐DA)	+	*B* = −0.13, *SE* = 0.06, *β* = −0.21	Multivariate, multiple regression
				Psychiatric symptoms: social impairment (ASD‐DA)	NS	*B* = 0.05, *SE* = 0.03, *β* = 0.18	Multivariate, multiple regression
				Psychiatric symptoms: restricted/repetitive behaviour (ASD‐DA)	NS	*B* = 0.05, *SE* = 0.06, *β* = 0.09	Multivariate, multiple regression
Matson et al. (2009), USA High quality	*n* = 257 adults (139M, 118F) with severe or profound intellectual disability, living in a developmental centre (*M*_age_ = 49.78, *SD* = 11.83, range 20–81 years)	Informant reports by care staff	Self‐injurious behaviour (ASD‐BPA)	Social skills: general positive social skills (MESSIER)	−	*B* = −0.01, *SE* = 0.00, *β* = −0.54	Multivariate, multiple regression
				Social skills: general negative social skills (MESSIER)	NS	*B* = 0.01, *SE* = 0.01, *β* = 0.20	Multivariate, multiple regression
			Aggressive behaviour in general (ASD‐BPA)	Social skills: general positive social skills (MESSIER)	−	*B* = −0.04, *SE* = 0.01, *β* = −0.62	Multivariate, multiple regression
				Social skills: general negative social skills (MESSIER)	+	*B* = 0.11, *SE* = 0.03, *β* = 0.61	Multivariate, multiple regression
Nøttestad and Linaker (2002), Norway Low quality	*n* = 22 adults with mild, moderate, severe, or profound intellectual disability, displaying physically aggressive behaviour (*M* = 37, range 22–75) *n* = 41 controls with intellectual disability (*M*_age_ = 44, range 22–75 years) and living in different settings	Informant reports by caretakers	Physically aggressive behaviour (caretaker reports: participant attacked people in the previous year)	Aggressive behaviour: destructive (caretaker reports: attacks on objects/property in the previous year)	+	Not reported	Univariate, Mann–Whitney *U*‐test
				Aggressive behaviour: self‐injurious (caretaker reports: behaviour not specified)	+	Not reported	Univariate, Mann–Whitney *U*‐test
			Destructive behaviour (caretaker reports: attacks on property in the previous year)	Aggressive behaviour: physical (caretaker reports)	+	Not reported	Univariate, Mann–Whitney *U*‐test
			Self‐injurious behaviour (caretaker reports: behaviour not specified)	Aggressive behaviour: physical (caretaker reports: attacks on people in the previous year)	+	Not reported	Univariate, Mann–Whitney *U*‐test
Novaco and Taylor (2004), UK High quality	129 male adults with intellectual disability residing in a forensic service (*M*_age_ = 33.2, *SD* = 11.6)	Self‐reports + retrospective case review + informant ratings by staff	Physically aggressive behaviour (case file: defined as an act that resulted in or could potentially have resulted in physical injury, displayed since admission)	Personality type: psychoticism (EPQ‐R Short Scale)	NS	*B* = 0.0121, *SE* = 0.019, *β* = 0.064, *t* = 0.63	Multivariate, hierarchical regression
				Personality type: neuroticism (EPQ‐R Short Scale)	NS	*B* = 0.0114, *SE* = 0.008, *β* = 0.132, *t* = 1.35	Multivariate, hierarchical regression
				Personality type: lie (EPQ‐R Short Scale)	NS	*B* = −0.0122, *SE* = 0.010, *β* = −0.125, *t* = 1.28	Multivariate, hierarchical regression
				Personality type: extraversion (EPQ‐R Short Scale)	+	*B* = 0.0245, *SE* = 0.010, *β* = 0.237, *t* = 2.55	Multivariate, hierarchical regression
				Self‐reported anger (NAS, PI, STAXI State Anger)	+	*NAS* *B* = 0.0078, *SE* = 003, *β* = 0.381, *t* = 3.08	Multivariate, hierarchical regression
					NS	*PI* *B* = −0.0018, *SE* = 0.002, *β* = −0.085, *t* = 0.74	Multivariate, hierarchical regression
					NS	*STAXI* *B* = −0.0129, *SE* = 0.008, *β* = −0.150, *t* = 1.55	Multivariate, hierarchical regression
Owen et al. (2004), UK Low quality	*n* = 93 adults (61M, 32F) with intellectual disability living in a long‐stay residential hospital (*M*_age_ = 55.2, *SD* = 12.7, range 24–93 years)	Informant reports by care staff	Self‐injurious behaviour (BPI)	Life events: negative life events (LEL)	NS	*r*(93) = .09	Univariate, Pearson correlation
			Aggressive behaviour in general (BPI)	Life events: negative life events (LEL)	+	*r*(88) = .27	Multivariate, Pearson partial correlation
Phillips and Rose (2010), UK Low quality	*n* = 20 adults (15M, 5F) with mild intellectual disability and challenging behaviour experiencing placement breakdown (*M*_age_ = 47.9, range 25.3–65.7 years) *n* = 23 adults (17M, 6F) with mild intellectual disability and challenging behaviour, that did not experience placement breakdown (*M*_age_ = 43.2, range 22.7–79.2 years). All participants were living in residential facilities	Informant reports by care staff	Physically aggressive behaviour (DAS‐B)	Life events: moves between community services (informant reports)	NS	OR = 1.19, CI [0.23; 6.11]	Univariate, odds ratio^a^
Rojahn et al. (2004), USA Low quality	*n* = 180 adults (97M, 83F) with mild, moderate, severe, or profound intellectual disability residing at a developmental centre (*M*_age_ = 50.6, *SD* = 14.5, range 20–91 years)	Informant reports by care staff	Self‐injurious behaviour (BPI)	Aggressive behaviour: general (BPI)	+	*ρ* = .25	Univariate, Spearman rank correlation
				Psychiatric symptoms: mania (DASH‐II)	+	*ρ* = .18	Univariate, Spearman rank correlation
				Psychiatric symptoms: PDD/autism (DASH‐II)	+	*ρ* = .19	Univariate, Spearman rank correlation
				Psychiatric symptoms: stereotypies/tics (DASH‐II)	+	*ρ* = .19	Univariate, Spearman rank correlation
				Psychiatric symptoms: organic syndromes (DASH‐II)	+	*ρ* = .24	Univariate, Spearman rank correlation
				Psychiatric symptoms: impulse control (DASH‐II)	+	*ρ* = .17	Univariate, Spearman rank correlation
				Psychiatric symptoms: self‐injurious behaviour (DASH‐II)	+	*ρ* = .27	Univariate, Spearman rank correlation
				Psychiatric symptoms: eating disorder (DASH‐II)	+	*ρ* = .15	Univariate, Spearman rank correlation
				Psychiatric symptoms: sexual disorder (DASH‐II)	+	*ρ* = .18	Univariate, Spearman rank correlation
				Psychiatric symptoms: total (DASH‐II)	+	*ρ* = .27	Univariate, Spearman rank correlation
				Psychiatric symptoms: anxiety (DASH‐II)	NS	Not reported	Univariate, Spearman rank correlation
				Psychiatric symptoms: schizophrenia (DASH‐II)	NS	Not reported	Univariate, Spearman rank correlation
				Psychiatric symptoms: elimination disorder (DASH‐II)	NS	Not reported	Univariate, Spearman rank correlation
				Psychiatric symptoms: sleep disorder (DASH‐II)	NS	Not reported	Univariate, Spearman rank correlation
			Aggressive behaviour in general (BPI)	Aggressive behaviour: self‐injurious (BPI)	+	*ρ *= .25	Univariate, Spearman rank correlation
				Psychiatric symptoms: total (DASH‐II)	+	*ρ* = .25	Univariate, Spearman rank correlation
				Psychiatric symptoms: depression (DASH‐II)	+	*ρ* = .16	Univariate, Spearman rank correlation
				Psychiatric symptoms: mania (DASH‐II)	+	*ρ* = .20	Univariate, Spearman rank correlation
				Psychiatric symptoms: impulse control (DASH‐II)	+	*ρ* = .33	Univariate, Spearman rank correlation
				Psychiatric symptoms: self‐injurious behaviour (DASH‐II)	+	*ρ* = .25	Univariate, Spearman rank correlation
				Psychiatric symptoms: anxiety (DASH‐II)	NS	Not reported	Univariate, Spearman rank correlation
				Psychiatric symptoms: PDD/autism(DASH‐II)	NS	Not reported	Univariate, Spearman rank correlation
				Psychiatric symptoms: schizophrenia (DASH‐II)	NS	Not reported	Univariate, Spearman rank correlation
				Psychiatric symptoms: stereotypies/tics (DASH‐II)	NS	Not reported	Univariate, Spearman rank correlation
				Psychiatric symptoms: organic syndromes (DASH‐II)	NS	Not reported	Univariate, Spearman rank correlation
				Psychiatric symptoms: elimination disorder (DASH‐II)	NS	Not reported	Univariate, Spearman rank correlation
				Psychiatric symptoms: eating disorder (DASH‐II)	NS	Not reported	Univariate, Spearman rank correlation
				Psychiatric symptoms: sleep disorder (DASH‐II)	NS	Not reported	Univariate, Spearman rank correlation
				Psychiatric symptoms: sexual disorder (DASH‐II)	NS	Not reported	Univariate, Spearman rank correlation
Rojahn et al. (2010), USA Low quality	*n* = 57 adults (38M, 19F) with mild, moderate, severe, or profound intellectual disability residing at a developmental centre (*M*_age_ = 50.98, *SD* = 11.55, range 23–81)	Informant reports by care staff	Self‐injurious behaviour (BPI‐01)	Psychiatric symptoms: ASD (ASD‐DA)	+	*F*(1, 55) = 6.32, *η* ^2^ = .10	Multivariate, ANOVA
			Self‐injurious behaviour (ASD‐BPA)		NS	Not reported	Multivariate, MANOVA
			Aggressive behaviour in general (BPI‐01, ASD‐BPA)	Psychiatric symptoms: ASD (ASD‐DA)	NS	*F*(1, 55) = 0.34, *η* ^2^ = .06	Multivariate, ANOVA
Ross and Oliver (2002), UK Low quality	*n* = 24 adults (15M, 9F) with severe or profound intellectual disability (*M*_age_ = 39.96, *SD* = 10.88)	Informant reports by care staff	Physically aggressive behaviour (CBI)	Psychiatric symptoms: mood, interest, pleasure (MIPQ)	NS	Not reported	Univariate, Fisher's Exact test
			Verbally aggressive behaviour (CBI)	Psychiatric symptoms: mood, interest, pleasure (MIPQ)	NS	Not reported	Univariate, Fisher's Exact test
			Destructive behaviour (CBI)	Psychiatric symptoms: mood, interest, pleasure (MIPQ)	NS	Not reported	Univariate, Fisher's Exact test
			Self‐injurious behaviour (CBI)	Psychiatric symptoms: mood, interest, pleasure (MIPQ)	NS	Not reported	Univariate, *χ* ^2^‐test
Sappok et al. (2014), Germany High quality	*n* = 203 adult patients of a psychiatric department (139M, 64F), with mild, moderate, or severe/profound intellectual disability (*M*_age_ = 35.8, *SD* = 12.6) and living in different settings	Retrospective chart review	Physically aggressive behaviour (MOAS)	Social skills: emotional development (SAED)	NS	Not reported	Univariate, Pearson correlation
				Psychiatric diagnosis: schizophrenia, mood disorders, neurotic disorders, personality disorders, ASD (ICD‐10 diagnosis as derived from case file)	NS	Not reported	Univariate, Pearson correlation
				Psychiatric diagnosis: dependency disorder (ICD‐10 diagnosis as derived from case file)	NS	*r* = .19	Univariate, Pearson correlation
			Verbally aggressive behaviour (MOAS)	Social skills: emotional development (SAED)	+	*β* = 0.26, CI [0.10; 0.43]	Multivariate, regression analysis
				Psychiatric diagnosis: schizophrenia (ICD‐10 diagnosis as derived from case file)	NS	*r* = −.19	Univariate, Pearson correlation
				Psychiatric diagnosis: mood disorders (ICD‐10 diagnosis as derived from case file)	NS	*r* = .17	Univariate, Pearson correlation
				Psychiatric diagnosis: neurotic disorders, ASD, dependency disorders (ICD‐10 diagnosis as derived from case file)	NS	Not reported	Univariate, Pearson correlation
				Psychiatric diagnosis: personality disorder (ICD‐10 diagnosis as derived from case file)	+	*β* = 1.05, CI [0.34; 1.76]	Multivariate, regression analysis
			Destructive behaviour (MOAS)	Social skills: emotional development (SAED)	NS	Not reported	Univariate, Pearson correlation
				Psychiatric diagnosis: schizophrenia, mood disorders, neurotic disorders, personality disorders, ASD, dependency disorders (ICD‐10 diagnosis as derived from case file)	NS	Not reported	Univariate, Pearson correlation
			Self‐injurious behaviour (MOAS)	Social skills: emotional development (SAED)	−	*β* = −0.38, CI [−0.53; −0.23]	Multivariate, regression analysis
				Psychiatric diagnosis: schizophrenia, mood disorders, neurotic disorders, personality disorders (ICD‐10 diagnosis as derived from case file)	NS	Not reported	Univariate, Pearson correlation
				Psychiatric diagnosis: dependency disorders (ICD‐10 diagnosis as derived from case file)	NS	*r* = .15	Univariate, Pearson correlation
				Psychiatric diagnosis: ASD (ICD‐10 diagnosis as derived from case file)	+	*β* = 0.49, CI [0.17; 0.80]	Multivariate, regression analysis
			Aggressive behaviour in general (MOAS)	Social skills: emotional development (SAED)	NS	Not reported	Univariate, Pearson correlation
				Psychiatric diagnosis: schizophrenia, mood disorders, neurotic disorders, personality disorders, ASD, dependency disorders (ICD‐10 diagnosis as derived from case file)	NS	Not reported	Univariate, Pearson correlation
Tenneij et al. (2009), the Netherlands High quality	*n* = 108 adults (82M, 26F) with mild intellectual disability residing in inpatient treatment facilities for individuals with severe behavioural and emotional problems (*M*_age_ = 26.4, *SD* = 7.5)	Informant reports by care staff	Aggressive behaviour in general (SOAS‐R)	Aggressive behaviour: self‐injurious (SOAS‐R)	+	OR = 6.2, CI [1; 38.9]	Multivariate, stepwise regression analysis
			Self‐injurious behaviour (SOAS‐R)	Aggressive behaviour: general (SOAS‐R)	+	OR = 6.2, CI [1; 38.9]	Multivariate, stepwise regression analysis
Thorson et al. (2008), USA Low quality	*n* = 58 adults (19M, 39F) older than 21 years, with mild, moderate, severe, or profound intellectual disability residing in developmental centres	Informant reports by care staff	Self‐injurious behaviour (BPI)	Psychiatric diagnosis: any axis I disorder (DSM‐IV‐TR, DASH‐II)	NS	Not reported	Multivariate, MANOVA post hoc pairwise comparisons
				Psychiatric diagnosis: schizophrenia (DSM‐IV‐TR, DASH‐II)	NS	Not reported	Multivariate, MANOVA post hoc pairwise comparisons
			Aggressive behaviour in general (BPI)	Psychiatric diagnosis: any axis I disorder (DSM‐IV‐TR, DASH‐II)	NS	Not reported	Multivariate, MANOVA post hoc pairwise comparisons
				Psychiatric diagnosis: schizophrenia (DSM‐IV‐TR, DASH‐II)	NS	Not reported	Multivariate, MANOVA post hoc pairwise comparisons
Totsika et al. (2008), UK Low quality	*n* = 58 adults (36M, 22F) with moderate or severe intellectual disability, living in a long‐term residential facility (*M*_age_ = 45.26, *SD* = 12, range 23–83 years)	Informant reports by care staff	Physically aggressive behaviour (Individual Schedule)	Psychiatric diagnosis: any (Individual Schedule)	NS	OR = 2.57, CI [0.57; 11.69]	Univariate, odds ratio^a^
			Self‐injurious behaviour (Individual Schedule)	Psychiatric diagnosis: any (Individual Schedule)	NS	OR = 0.42, CI [0.12; 1.38]	Univariate, odds ratio^a^
Tsiouris et al. (2011), USA High quality	*n* = 4,069 adults (2,445M, 1,624F) with mild, moderate, severe, or profound intellectual disability living in the community and receiving services (*M*_age_ = 49.6, *SD* = 14.0)	Retrospective chart review + informant reports by care staff	Physically aggressive behaviour (IBR‐MOAS)	Psychiatric diagnosis: autism (DSM‐IV or DSM‐IV‐TR diagnosis derived from case file)	+	Incidence rate ratio = 1.285	Multivariate, incidence rate ratio^a^
				Psychiatric diagnosis: anxiety (DSM‐IV or DSM‐IV‐TR diagnosis derived from case file)	+	Incidence rate ratio = 1.121	Multivariate, incidence rate ratio^a^
				Psychiatric diagnosis: bipolar (DSM‐IV or DSM‐IV‐TR diagnosis derived from case file)	+	Incidence rate ratio = 1.560	Multivariate, incidence rate ratio^a^
				Psychiatric diagnosis: psychosis (DSM‐IV or DSM‐IV‐TR diagnosis derived from case file)	+	Incidence rate ratio = 1.477	Multivariate, incidence rate ratio^a^
				Psychiatric diagnosis: impulse control disorder (DSM‐IV or DSM‐IV‐TR diagnosis derived from case file)	+	Incidence rate ratio = 1.752	Multivariate, incidence rate ratio^a^
				Psychiatric diagnosis: personality (DSM‐IV or DSM‐IV‐TR diagnosis derived from case file)	+	Incidence rate ratio = 1.271	Multivariate, incidence rate ratio^a^
				Psychiatric diagnosis: OCD (DSM‐IV or DSM‐IV‐TR diagnosis derived from case file)	NS	Incidence rate ratio = 1.132	Multivariate, incidence rate ratio^a^
				Psychiatric diagnosis: depression (DSM‐IV or DSM‐IV‐TR diagnosis derived from case file)	NS	Incidence rate ratio = 1.093	Multivariate, incidence rate ratio^a^
			Verbally aggressive behaviour (against self and against others) (IBR‐MOAS)	Psychiatric diagnosis: depression (DSM‐IV or DSM‐IV‐TR diagnosis derived from case file)	+	*Against self* Incidence rate ratio = 1.271 *Against others* Incidence rate ratio = 1.154	Multivariate, incidence rate ratio^a^
				Psychiatric diagnosis: bipolar (DSM‐IV or DSM‐IV‐TR diagnosis derived from case file)	+	*Against self* Incidence rate ratio = 1.292 *Against others* Incidence rate ratio = 1.402	Multivariate, incidence rate ratio^a^
				Psychiatric diagnosis: psychosis (DSM‐IV or DSM‐IV‐TR diagnosis derived from case file)	+	*Against self* Incidence rate ratio = 1.388 *Against others* Incidence rate ratio = 1.322	Multivariate, incidence rate ratio^a^
				Psychiatric diagnosis: impulse control disorder (DSM‐IV or DSM‐IV‐TR diagnosis derived from case file)	+	*Against self* Incidence rate ratio 1.401 *Against others* Incidence rate ratio = 1.560	Multivariate, incidence rate ratio^a^
				Psychiatric diagnosis: personality (DSM‐IV or DSM‐IV‐TR diagnosis derived from case file)	+	*Against self* Incidence rate ratio = 1.422 *Against others* Incidence rate ratio = 1.257	Multivariate, incidence rate ratio^a^
				Psychiatric diagnosis: anxiety (DSM‐IV or DSM‐IV‐TR diagnosis derived from case file)	+	*Against self* Incidence rate ratio = 1.208	Multivariate, incidence rate ratio^a^
					NS	*Against others* Incidence rate ratio = 1.083	Multivariate, incidence rate ratio^a^
				Psychiatric diagnosis: autism (DSM‐IV or DSM‐IV‐TR diagnosis derived from case file)	NS	*Against self* Incidence rate ratio = 1.014 *Against others* Incidence ratio = 0.925	Multivariate, incidence rate ratio^a^
				Psychiatric diagnosis: OCD (DSM‐IV or DSM‐IV‐TR diagnosis derived from case file)	NS	*Against self* Incidence rate ratio = 1.099 *Against others* Incidence rate ratio = 1.055	Multivariate, incidence rate ratio^a^
			Destructive behaviour (IBR‐MOAS)	Psychiatric diagnosis: autism (DSM‐IV or DSM‐IV‐TR diagnosis derived from case file)	+	Incidence rate ratio = 1.257	Multivariate, incidence rate ratio^a^
				Psychiatric diagnosis: anxiety (DSM‐IV or DSM‐IV‐TR diagnosis derived from case file)	+	Incidence rate ratio = 1.200	Multivariate, incidence rate ratio^a^
				Psychiatric diagnosis: OCD (DSM‐IV or DSM‐IV‐TR diagnosis derived from case file)	+	Incidence rate ratio = 1.232	Multivariate, incidence rate ratio^a^
				Psychiatric diagnosis: bipolar (DSM‐IV or DSM‐IV‐TR diagnosis derived from case file)	+	Incidence rate ratio = 1.517	Multivariate, incidence rate ratio^a^
				Psychiatric diagnosis: psychosis (DSM‐IV or DSM‐IV‐TR diagnosis derived from case file)	+	Incidence rate ratio = 1.294	Multivariate, incidence rate ratio^a^
				Psychiatric diagnosis: impulse control disorder (DSM‐IV or DSM‐IV‐TR diagnosis derived from case file)	+	Incidence rate ratio = 1.839	Multivariate, incidence rate ratio^a^
				Psychiatric diagnosis: personality (DSM‐IV or DSM‐IV‐TR diagnosis derived from case file)	+	Incidence rate ratio = 1.300	Multivariate, incidence rate ratio^a^
				Psychiatric diagnosis: depression (DSM‐IV or DSM‐IV‐TR diagnosis derived from case file)	NS	Incidence rate ratio = 1.051	Multivariate, incidence rate ratio^a^
			Self‐injurious behaviour (IBR‐MOAS)	Psychiatric diagnosis: ASD (DSM‐IV or DSM‐IV‐TR diagnosis derived from case file)	+	Incidence rate ratio = 1.383	Multivariate, incidence rate ratio^a^
				Psychiatric diagnosis: anxiety (DSM‐IV or DSM‐IV‐TR diagnosis derived from case file)	+	Incidence rate ratio = 1.343	Multivariate, incidence rate ratio^a^
				Psychiatric diagnosis: bipolar (DSM‐IV or DSM‐IV‐TR diagnosis derived from case file)	+	Incidence rate ratio = 1.495	Multivariate, incidence rate ratio^a^
				Psychiatric diagnosis: psychosis (DSM‐IV or DSM‐IV‐TR diagnosis derived from case file)	+	Incidence rate ratio = 1.176	Multivariate, incidence rate ratio^a^
				Psychiatric diagnosis: impulse control disorder (DSM‐IV or DSM‐IV‐TR diagnosis derived from case file)	+	Incidence rate ratio = 1.664	Multivariate, incidence rate ratio^a^
				Psychiatric diagnosis: personality (DSM‐IV or DSM‐IV‐TR diagnosis derived from case file)	+	Incidence rate ratio = 1.598	Multivariate, incidence rate ratio^a^
				Psychiatric diagnosis: depression (DSM‐IV or DSM‐IV‐TR diagnosis derived from case file)	NS	Incidence rate ratio = 1.126	Multivariate, incidence rate ratio^a^
				Psychiatric diagnosis: OCD (DSM‐IV or DSM‐IV‐TR diagnosis derived from case file)	NS	Incidence rate ratio = 1.190	Multivariate, incidence rate ratio^a^
Turygin et al. (2013), USA Low quality	*n* = 332 adults (180M, 152F) with mild, moderate, severe, or profound intellectual disability residing in a developmental centre	Informant reports by	Self‐injurious behaviour (ASD‐BPA)	Psychiatric symptoms: depression (DASH‐II depression subscale)	+	*r* = .15, CI [−0.01; 0.30]	Univariate, Pearson correlation
			Aggressive behaviour in general (ASD‐BPA)	Psychiatric symptoms: depression (DASH‐II depression subscale)	+	*r* = .40, CI [.26; 0.52]	Univariate, Pearson correlation
Tyrer et al. (2006), UK Low quality	*n* = 3,062 adults (1,745M, 1,317F) with mild, moderate, severe or profound intellectual disability living in Leicester (range 19–92 years) and living in different settings	Retrospective case review	Physically aggressive behaviour (case file: defined as physically aggressive behaviour towards others that occurred within the last 12 months and that was rated by a carer as either frequent or severe, or both frequent and severe)	Living situation: residential home (compared to independent living) (case file)	+	OR = 2.79, CI [1.55; 5.02]	Multivariate, logistic regression
				Living situation: NHS accommodation (compared to independent living) (case file)	+	OR = 4.90, CI [2.52; 9.52]	Multivariate, logistic regression
				Living situation: living with relatives (compared to independent living) (case file)	+	OR = 1.11, CI [0.61; 2.01]	Multivariate, logistic regression
				Living situation: other (compared to independent living) (case file)	+	OR = 1.22, CI [0.24; 6.08]	Multivariate, logistic regression
				Psychiatric diagnosis: ASD (case file)	NS	OR = 1.32, CI [0.74; 2.35]	Multivariate, logistic regression
				Psychiatric symptoms: frustration (case file: carers were asked whether the symptom had been present recently)	+	*Minor* OR = 0.90–1.79 *Major* OR = 2.15–4.44	Multivariate, logistic regression
				Psychiatric symptoms: mood swings (case file: carers were asked whether the symptom had been present recently)	+	*Minor* OR = 1.34–2.73 *Major* OR = 6.43–13.40	Multivariate, logistic regression
				Psychiatric symptoms: unhappiness/upset/crying (case file: carers were asked whether the symptom had been present recently)	NS	*Minor* OR = 0.85–1.60 *Major* OR = 0.94–2.19	Multivariate, logistic regression
				Psychiatric symptoms: withdrawal (case file: carers were asked whether the symptom had been present recently)	NS	*Minor* OR = 0.82–1.54 *Major* OR = 0.60–1.50	Multivariate, logistic regression
				Psychiatric symptoms: anxiousness/phobias/irrational fears (case file: carers were asked whether the symptom had been present recently)	NS	*Minor* OR = 0.72–1.38 *Major* OR = 0.85–1.72	Multivariate, logistic regression
				Psychiatric symptoms: feeling things always set against them (case file: carers were asked whether the symptom had been present recently)	NS	*Minor* OR = 0.67–1.46 *Major* OR = 0.56–1.46	Multivariate, logistic regression
				Psychiatric symptoms: lethargy (case file: carers were asked whether the symptom had been present recently)	NS	*Minor* OR = 0.64–1.23 *Major* OR = 0.63–1.48	Multivariate, logistic regression

Abbreviations: ABC, Aberrant Behaviour Checklist; ABCL, Adult Behaviour Checklist; AQC, Attachment Questionnaire for Children; ASD‐BPA, Autism Spectrum Disorder‐Behaviour Problems for Adults; ASD‐DA, Autism Spectrum Disorder‐Diagnosis for intellectually disabled adults; BPI, Behaviour Problems Inventory; BPI‐S, Behaviour Problems Inventory Short Form; CBI, Challenging Behaviour Interview; CCB, Checklist of Challenging Behaviour; DAS, Disability Assessment Schedule; DAS‐B, Disability Assessment Schedule for behaviour problems; DASH‐II, Diagnostic Assessment of the Severely Handicapped‐II; DBC‐A, Developmental Behaviour Checklist for Adults; DSM, Diagnostic and Statistical Manual of Mental Disorders; EPQ‐R, Eysenck Personality Questionnaire‐Revised; IBR‐MOAS, Institute for Basic Research‐Modified Overt Aggression Scale; ICAP, Inventory for Client and Agency Planning; ICD, International Classification of Diseases and Related Health Problems; LEL, Life Events List; LES, Life Event Scale; MESSIER, Matson Evaluation of Social skills in Individuals with Severe Retardation; MIPQ, Mood, Interest and Pleasure Questionnaire; MIPQ‐S, Mood, Interest and Pleasure Questionnaire‐Short Form; MOAS(+), Modified Over Aggression Scale; NAS, Novaco Anger Scale; OAS, Observer Alexithymia Scale; PAS‐ADD, Psychiatric Assessment Schedule for Adults with Developmental Disabilities; PI, Provocation Inventory; PIMRA, Psychopathology Instrument for Mentally Retarded Adults; RSMB, Reiss Screen for Maladaptive Behaviour; SF‐36, Short Form Health Survey; SIB‐R, Scales of Independent Behaviour‐Revised; SOAS‐R, Staff Observation Aggression Scale‐Revised; STAXI, Spielberger State‐Trait Anger Expression Inventory; TESI, Traumatic Events Screening Inventory; WARS, Ward Anger Rating Scale.

^a^ Odds ratio or incidence rate ratio calculated based on information reported in the study.

^b^ For the purpose of correctly interpreting results, the direction of this association was changed.

**TABLE 2:** Associations with physically aggressive behaviour

Factor	Positive association	No association	Negative association
Behavioural factors			
Aggressive behaviour			
Verbally aggressive behaviour	Crocker et al., (2006), Drieschner et al. (2013)		
Destructive behaviour	Crocker et al. (2006), Drieschner et al., (2013), Nøttestad and Linaker (2002)		
Sexually aggressive behaviour	Crocker et al. (2006), Drieschner et al. (2013)		
Self‐injurious behaviour	Crocker et al. (2006), Drieschner et al. (2013), Nøttestad and Linaker (2002)	**Bernstein et al. (** **2015** **)**	
Aggressive behaviour in general	**Bernstein et al. (** **2015** **)**		
Criminal behaviour	Crocker et al. (2006)	Lunsky et al. (2012)	Drieschner et al. (2013)
Psychiatric disorders and symptoms			
Psychiatric disorders			
Substance use disorders (F10–F19)		Drieschner et al. (2013), Lunsky et al. (2012) **Sappok et al. (** **2014** **)**	
Schizophrenia and delusional disorders (F20–F29)	**Tsiouris et al. (** **2011** **)**	Drieschner et al. (2013), **Sappok et al. (** **2014** **)**	
Mood disorders (F30–F39)	**Tsiouris et al. (** **2011** **)**	Drieschner et al. (2013), **Sappok et al. (** **2014** **)**, **Tsiouris et al. (** **2011** **)**	
Neurotic, stress‐related and somatoform disorders (F40–F48)	**Tsiouris et al. (** **2011** **)**	Drieschner et al. (2013), **Sappok et al. (** **2014** **)**, **Tsiouris et al. (** **2011** **)**	
Personality disorders (F60–F69)	**Tsiouris et al. (** **2011** **)**	Alexander et al. (2010), Drieschner et al. (2013), **Sappok et al. (** **2014** **)**	
Disorders of psychological development (F80–F89)	**Tsiouris et al. (** **2011** **)**	Drieschner et al. (2013), Lunsky et al. (2012), **Sappok et al. (** **2014** **)**, Tyrer et al. (2006)	
Behavioural and emotional disorders with onset in childhood/adolescence (F90–F98)	Drieschner et al. (2013), Lindsay et al. (2013)		
Any psychiatric diagnosis		Totsika et al. (2008)	
Number of psychiatric diagnoses		**Crocker et al. (** **2014** **)**	
Severity of psychiatric diagnoses		**Crocker et al. (** **2014** **)**	
Psychiatric symptoms			
Symptoms of mood disorders (F30–F39)		**Bernstein et al. (** **2015** **)**, Ross and Oliver (2002)	
Aspecific psychiatric symptoms	Tyrer et al. (2006)^a^	Tyrer et al. (2006)^b^	
Psychosocial factors			
Adaptive skills			**Hartley and MacLean (** **2007** **)**
Anger	**Novaco and Taylor (** **2004** **)**	**Novaco and Taylor (** **2004** **)**	
Life events		Lunsky et al. (2012), Phillips and Rose (2010)	
Living situation			
Group home		Crocker et al. (2006)	
Independent		Crocker et al. (2006)	Lunsky et al. (2012)
Institution	Tyrer et al. (2006)^c^		
With family	Tyrer et al. (2006)^c^	Crocker et al. (2006), Lunsky et al. (2012)	
Other	Crocker et al. (2006), Tyrer et al. (2006)^c^		
Personality type	**Novaco and Taylor (** **2004** **)**	**Novaco and Taylor (** **2004** **)**	
Social skills			
Positive social skills		**Sappok et al. (** **2014** **)**	

Note

High‐quality studies are displayed in bold.

^a^ Frustration, mood swings.

^b^ Unhappiness/upset/crying, withdrawal, anxiousness/phobias/irrational fears, feeling things always set against them, lethargy.

^c^ Compared to independent living.

**TABLE 3:** Associations with verbally aggressive behaviour

Factor	Positive association	No association	Negative association
Behavioural factors			
Aggressive behaviour			
Physically aggressive behaviour	Crocker et al. (2006), Drieschner et al. (2013)		
Destructive behaviour	Crocker et al. (2006), Drieschner et al. (2013)		
Sexually aggressive behaviour	Crocker et al. (2006), Drieschner et al. (2013)		
Self‐injurious behaviour	Crocker et al. (2006), Drieschner et al. (2013)		
Criminal behaviour	Crocker et al. (2006)		Drieschner et al. (2013)
Psychiatric disorders and symptoms			
Psychiatric disorders			
Substance use disorders (F10–F19)		Drieschner et al. (2013)	
Schizophrenia and delusional disorders (F20–F29)	**Tsiouris et al. (** **2011** **)**	Drieschner et al. (2013), **Sappok et al. (** **2014** **)**	
Mood disorders (F30–F39)	**Tsiouris et al. (** **2011** **)**	Drieschner et al. (2013), **Sappok et al. (** **2014** **)**	
Neurotic, stress‐related and somatoform disorders (F40–F48)	**Tsiouris et al. (** **2011** **)**	Drieschner et al. (2013), **Sappok et al. (** **2014** **)**, **Tsiouris et al. (** **2011** **)**	
Personality disorders (F60–F69)	**Sappok et al. (** **2014** **)**, **Tsiouris et al. (** **2011** **)**	Alexander et al. (2010), Drieschner et al. (2013)	
Disorders of psychological development (F80–F89)		Drieschner et al. (2013), **Tsiouris et al. (** **2011** **)**	
Behavioural and emotional disorders with onset in childhood/adolescence (F90–F98)	Drieschner et al. (2013)	Lindsay et al. (2013)	
Number of psychiatric diagnoses	**Crocker et al. (** **2014** **)**		
Severity of psychiatric diagnoses	**Crocker et al. (** **2014** **)**		
Psychiatric symptoms			
Symptoms of mood disorders (F30–F39)		Ross and Oliver (2002)	
Psychosocial factors			
Living situation			
Group home		Crocker et al. (2006)	
Independent		Crocker et al. (2006)	
With family		Crocker et al. (2006)	
Other	Crocker et al. (2006)		
Social skills			
Positive social skills	**Sappok et al. (** **2014** **)**		

Note

High‐quality studies are displayed in bold.

**TABLE 4:** Associations with destructive behaviour

Factor	Positive association	No association	Negative association
Behavioural factors			
Aggressive behaviour			
Physically aggressive behaviour	Crocker et al. (2006), Drieschner et al. (2013), Nøttestad and Linaker (2002)	Alexander et al. (2015)	
Verbally aggressive behaviour	Crocker et al. (2006), Drieschner et al. (2013)	Alexander et al. (2015)	
Destructive behaviour		Alexander et al. (2015)	
Sexually aggressive behaviour	Crocker et al. (2006), Drieschner et al. (2013)	Alexander et al. (2015)	
Self‐injurious behaviour	Crocker et al. (2006), Drieschner et al. (2013)	Alexander et al. (2015)	
Criminal behaviour	Alexander et al. (2015), Crocker et al. (2006), Lunsky et al. (2012)	Alexander et al. (2015)	Drieschner et al. (2013)
Psychiatric disorders and symptoms			
Psychiatric disorders			
Substance use disorders (F10–F19)		Alexander et al. (2015), **Sappok et al. (** **2014** **)**	Drieschner et al. (2013)
Schizophrenia and delusional disorders (F20–F29)	**Tsiouris et al. (** **2011** **)**	Alexander et al. (2015), Drieschner et al. (2013), **Sappok et al. (** **2014** **)**	
Mood disorders (F30–F39)	**Tsiouris et al. (** **2011** **)**	Alexander et al. (2015), Drieschner et al. (2013), **Sappok et al. (** **2014** **)**, **Tsiouris et al. (** **2011** **)**	
Neurotic, stress‐related and somatoform disorders (F40–F48)	**Tsiouris et al. (** **2011** **)**	Drieschner et al. (2013), **Sappok et al. (** **2014** **)**	
Personality disorders (F60–F69)	Alexander et al. (2015), **Tsiouris et al. (** **2011** **)**	Alexander et al. (2010), Drieschner et al. (2013), **Sappok et al. (** **2014** **)**, **2011**	
Disorders of psychological development (F80–F89)	**Tsiouris et al. (** **2011** **)**	Alexander et al. (2015), Drieschner et al. (2013), **Sappok et al. (** **2014** **)**	
Behavioural and emotional disorders with onset in childhood/adolescence (F90–F98)	Drieschner et al. (2013), Lindsay et al. (2013)		
Number of psychiatric diagnoses		**Crocker et al. (** **2014** **)**	
Severity of psychiatric diagnoses			**Crocker et al. (** **2014** **)**
Psychiatric symptoms			
Symptoms of organic mental disorders (F00–F09)	Allen et al. (2012)		
Symptoms of schizophrenia and delusional disorders (F20–F29)		Allen et al. (2012)	
Symptoms of mood disorders (F30–F39)	Allen et al. (2012)	Ross and Oliver (2002)	
Symptoms of neurotic, stress‐related and somatoform disorders (F40–F48)	Allen et al. (2012)		
Aspecific psychiatric symptoms	**Hemmings et al. (** **2006** **)** ^a^	**Hemmings et al. (** **2006** **)** ^b^	
Psychosocial factors			
Adaptive skills			**Hartley and MacLean (** **2007** **)**
Life events	Alexander et al. (2015)	Alexander et al. (2015)	
Living situation			
Group home		Crocker et al. (2006)	
Independent		Crocker et al. (2006)	
With family		Crocker et al. (2006)	
Other	Crocker et al. (2006)		
Social skills			
Positive social skills		**Sappok et al. (** **2014** **)**	**Hemmings et al. (** **2006** **)**

Note

High‐quality studies are displayed in bold.

^a^ Low energy, delayed sleep.

^b^ Anhedonia, sad or down, fearful/panicky, repetitive actions, too high or happy, suicidal, loss of appetite, weight change, loss of confidence, avoiding social contact, worthlessness, early waking, restlessness, irritable mood, loss of self‐care, odd language.

**TABLE 5:** Associations with sexually aggressive behaviour

Factor	Positive association	No association	Negative association
Behavioural factors			
Aggressive behaviour			
Physically aggressive behaviour	Crocker et al. (2006), Drieschner et al. (2013)		
Verbally aggressive behaviour	Crocker et al. (2006), Drieschner et al. (2013)		
Destructive behaviour	Crocker et al. (2006), Drieschner et al. (2013)		
Self‐injurious behaviour	Crocker et al. (2006), Drieschner et al. (2013)		
Criminal behaviour	Crocker et al. (2006)	Drieschner et al. (2013)	
Psychiatric disorders and symptoms			
Psychiatric disorders			
Substance use disorders (F10–F19)			Drieschner et al. (2013)
Schizophrenia and delusional disorders (F20–F29)		Drieschner et al. (2013)	
Mood disorders (F30–F39)		Drieschner et al. (2013)	
Neurotic, stress‐related and somatoform disorders (F40–F48)	**Crocker et al. (** **2014** **)**	Drieschner et al. (2013)	
Personality disorders (F60–F69)		Alexander et al. (2010), Drieschner et al. (2013)	
Disorders of psychological development (F80–F89)	**Cervantes and Matson (** **2015** **)**	Drieschner et al. (2013)	
Behavioural and emotional disorders with onset in childhood/adolescence (F90–F98)	Drieschner et al. (2013)	Lindsay et al. (2013)	
Psychosocial factors			
Living situation			
Group home		Crocker et al. (2006)	
Independent		Crocker et al. (2006)	
With family		Crocker et al. (2006)	
Other	Crocker et al. (2006)		

Note

High‐quality studies are displayed in bold.

**TABLE 6:** Associations with self‐injurious behaviour

Factor	Positive association	No association	Negative association
Behavioural factors			
Aggressive behaviour			
Physically aggressive behaviour	Clark et al. (2016), Drieschner et al. (2013), Nøttestad and Linaker (2002)	**Bernstein et al. (** **2015** **)**	
Verbally aggressive behaviour	Clark et al. (2016), Drieschner et al. (2013)		
Destructive behaviour	Clark et al. (2016), Drieschner et al. (2013)		
Sexually aggressive behaviour	Clark et al. (2016), Drieschner et al. (2013)		
Aggressive behaviour in general	**Bernstein et al. (** **2015** **)**, Bowring et al. (2017), Rojahn et al. (2004), **Tenneij et al. (** **2009** **)**		
Criminal behaviour	Lunsky et al. (2012)	Crocker et al. (2006)	Drieschner et al. (2013)
Psychiatric disorders and symptoms			
Psychiatric diagnosis			
Substance use disorders (F10–F19)		Drieschner et al. (2013), **Sappok et al. (** **2014** **)**	
Schizophrenia and delusional disorders (F20–F29)	**Tsiouris et al. (** **2011** **)**	Drieschner et al. (2013), **Sappok et al. (** **2014** **)**, Thorson et al. (2008)	
Mood disorders (F30–F39)	Hurley (2008), **Tsiouris et al. (** **2011** **)** ^a^	Drieschner et al. (2013), **Sappok et al. (** **2014** **)**, **Tsiouris et al. (** **2011** **)** ^b^	
Neurotic, stress‐related and somatoform disorders (F40–F48)	**Tsiouris et al. (** **2011** **)** ^c^	Drieschner et al. (2013), **Sappok et al. (** **2014** **)**, **Tsiouris et al. (** **2011** **)** ^d^	
Personality disorders (F60–F69)	Drieschner et al. (2013), **Tsiouris et al. (** **2011** **)**	Alexander et al. (2010), Drieschner et al. (2013), **Sappok et al. (** **2014** **)**	
Disorders of psychological development (F80–F89)	Cervantes and Matson (2015), **Horovitz et al. (** **2013** **)**, **Sappok et al. (** **2014** **)**, **Tsiouris et al. (** **2011** **)**	Bowring et al. (2017), Drieschner et al. (2013)	
Behavioural and emotional disorders with onset in childhood/adolescence (F90–F98)	Drieschner et al. (2013)		
Any psychiatric diagnosis		Bowring et al. (2017), Thorson et al. (2008), Totsika et al. (2008)	
Psychiatric symptoms			
Symptoms of organic mental disorders (F00–F09)	Rojahn et al. (2004)	Allen et al. (2012)	
Symptoms of schizophrenia and delusional disorders (F20–F29)	**Clark et al. (** **2016** **)**	Allen et al. (2012), Lundqvist (2013), Rojahn et al. (2004)	
Symptoms of mood disorders (F30–F39)	**Clark et al. (** **2016** **)**, Rojahn et al. (2004), Turygin et al. (2013)	Allen et al. (2012), **Bernstein et al. (** **2015** **)**, Lundqvist (2013), Ross and Oliver (2002)	
Symptoms of neurotic, stress‐related and somatoform disorders (F40–F48)		Allen et al. (2012), Lundqvist (2013), Rojahn et al. (2004)	
Symptoms of behavioural syndromes associated with physi(ologi)cal factors (F50–F59)	Rojahn et al. (2004, 2010)	Rojahn et al. (2004, 2010)	
Symptoms of personality disorders (F60‐F69)	**Clark et al. (** **2016** **)**, Rojahn et al. (2004)		
Symptoms of disorders of psychological development (F80–F89)	Lundqvist (2013), **Matson & Rivet (** **2008** **)**, Rojahn et al. (2004)	**Matson and Rivet (** **2008** **)**	
Symptoms of behavioural and emotional disorders with onset in childhood/adolescence (F90–F98)		Larson et al. (2011), Lundqvist (2013), Rojahn et al. (2004)	
Total psychiatric symptoms	**Clark et al. (** **2016** **)**, Rojahn et al. (2004)	Lundqvist (2013)	
Aspecific psychiatric symptoms	**Hemmings et al. (** **2006** **)** ^e^, Rojahn et al. (2004)^f^	**Hemmings et al. (** **2006** **)** ^g^	
Psychosocial factors			
Adaptive skills	Bowring et al. (2017)^h^		
Communication skills	Bowring et al. (2017)^h^, Lundqvist (2013)^i^	Lundqvist (2013)^j^	
Life events	**Clark et al. (** **2016** **)**	Owen et al. (2004)	
Living situation			
Group home		Crocker et al. (2006)	
Independent		Crocker et al. (2006)	
With family		Bowring et al. (2017), Crocker et al. (2006)	
Other	Bowring et al. (2017)^k^, Crocker et al. (2006)		
Social skills			
Positive social skills		**Hemmings et al. (** **2006** **)**, Lundqvist (2013)	**Matson et al. (** **2009** **)**, **Sappok et al. (** **2014** **)**
Negative social skills		**Matson et al. (** **2009** **)**	

Note

High‐quality studies are displayed in bold.

^a^ Bipolar.

^b^ Depression.

^c^ Anxiety.

^d^ OCD.

^e^ Irritable mood, suicidal.

^f^ Stereotypies/tics, impulse control, self‐injury.

^g^ Low energy, anhedonia, sad or down, fearful/panicky, repetitive actions, too high or happy, loss of appetite, weight change, loss of confidence, avoiding social contact, worthlessness, delayed sleep, early waking, restlessness, loss of self‐care, odd language.

^h^ For the purpose of correctly interpreting results, the direction of these associations was changed.

^i^ Communicating with pictures.

^j^ Communicating with writing, speech, signs, gestures and sounds.

^k^ Paid care.

**TABLE 7:** Associations with aggressive behaviour in general

Factor	Positive association	No association	Negative association
Psychosocial factors			
Aggressive behaviour			
Physically aggressive behaviour	**Bernstein et al. (** **2015** **)**		
Self‐injurious behaviour	**Bernstein et al. (** **2015** **)**, Bowring et al. (2017), Rojahn et al. (2004), **Tenneij et al. (** **2009** **)**		
Learned function of aggressive behaviour		**Koritsas and Iacono (** **2015** **)**	
Criminal behaviour	Crocker et al. (2006)		Drieschner et al. (2013)
Psychiatric disorders and symptoms			
Psychiatric diagnosis			
Substance use disorders (F10–F19)		**Sappok et al. (** **2014** **)**	Drieschner et al. (2013)
Schizophrenia and delusional disorders (F20–F29)		Drieschner et al. (2013), **Sappok et al. (** **2014** **)**, Thorson et al. (2008)	
Mood disorders (F30–F39)	Hurley (2008)	Drieschner et al. (2013), **Sappok et al. (** **2014** **)**	
Neurotic, stress‐related and somatoform disorders (F40–F48)		Drieschner et al. (2013), **Sappok et al. (** **2014** **)**	
Personality disorders (F60–F69)		Drieschner et al. (2013), **Sappok et al. (** **2014** **)**	
Disorders of psychological development (F80–F89)	Bowring et al. (2017)	Drieschner et al. (2013), **Horovitz et al. (** **2013** **)**, **Sappok et al. (** **2014** **)**	
Behavioural and emotional disorders with onset in childhood/adolescence (F90–F98)	Drieschner et al. (2013)		
Any psychiatric diagnosis		Bowring et al. (2017), Thorson et al. (2008)	
Psychiatric symptoms			
Symptoms of organic mental disorders (F00–F09)	Allen et al. (2012)	Rojahn et al. (2004)	
Symptoms of substance use disorders (F10–F19)	Didden et al. (2009)		
Symptoms of schizophrenia and delusional disorders (F20–F29)	**Clark et al. (** **2016** **)**	Allen et al. (2012), Lundqvist (2013), Rojahn et al. (2004)	
Symptoms of mood disorders (F30–F39)	Allen et al. (2012), Rojahn et al. (2004), Turygin et al. (2013)	**Bernstein et al. (** **2015** **)**, **Clark et al. (** **2016** **)**, **Koritsas and Iacono (** **2015** **)**, Lundqvist (2013)	
Symptoms of neurotic, stress‐related and somatoform disorders (F40–F48)	Allen et al. (2012), **Koritsas and Iacono (** **2015** **)**	Lundqvist (2013), Rojahn et al. (2004)	
Symptoms of behavioural syndromes associated with physi(ologi)cal factors (F50–F59)		Rojahn et al. (2004)	
Symptoms of personality disorders (F60–F69)	**Clark et al. (** **2016** **)**	Rojahn et al. (2004)	
Symptoms of disorders of psychological development (F80–F89)	**Davies et al. (** **2015** **)**, Lundqvist (2013), **Matson and Rivet (** **2008** **)** ^a^	**Davies et al. (** **2015** **)**, **Matson and Rivet (** **2008** **)** ^b^, Rojahn et al. (2004, 2010)	
Symptoms of behavioural and emotional disorders with onset in childhood/adolescence (F90–F98)		Larson et al. (2011), Lundqvist (2013), Rojahn et al. (2004)	
Total psychiatric symptoms	**Clark et al. (** **2016** **)**, **Koritsas and Iacono (** **2015** **)**, Rojahn et al. (2004)	Lundqvist (2013)	
Aspecific psychiatric symptoms	**Hemmings et al. (** **2006** **)** ^c^, **Koritsas and Iacono (** **2015** **)** ^d^, Rojahn et al. (2004)^e^	**Hemmings et al. (** **2006** **)** ^f^, **Koritsas and Iacono (** **2015** **)** ^g^, Rojahn et al. (2004)^h^	
Psychosocial factors			
Communication skills	Bowring et al. (2017)^i^, Lundqvist (2013)^j^	Bowring et al. (2017)^k^, **Koritsas and Iacono (** **2015** **)**, Lundqvist (2013)^l^	
Life events	**Clark et al. (** **2016** **)**, **Esbensen and Benson (** **2006** **)**, Owen et al. (2004)	**Esbensen and Benson (** **2006** **)**	
Living situation			
Group home		Crocker et al. (2006)	
Independent		Crocker et al. (2006)	
With family		Bowring et al. (2017), Crocker et al. (2006), **Koritsas and Iacono (** **2015** **)** ^m^	
Other	Crocker et al. (2006)	Bowring et al. (2017)	
Social skills			
Positive social skills	Lundqvist (2013)	**Hemmings et al. (** **2006** **)**, **Sappok et al. (** **2014** **)**	Lundqvist (2013), **Matson et al. (** **2009** **)**
Negative social skills	**Matson et al. (** **2009** **)**		

Note

High‐quality studies are displayed in bold.

^a^ Communication impairment associated with ASD.

^b^ Social impairment and restricted/repetitive behaviour associated with ASD.

^c^ Early waking, low energy, irritable mood.

^d^ Disruption.

^e^ Impulse control, self‐injurious behaviour.

^f^ Anhedonia, sad or down, fearful/panicky, repetitive actions, too high or happy, suicidal, loss of appetite, weight change, loss of confidence, avoiding social contact, worthlessness, delayed sleep, restlessness, loss of self‐care, odd language.

^g^ Self‐absorbed, communication disturbance, social relating.

^h^ Stereotypies/tics.

^i^ Understanding, being verbal; for the purpose of correctly interpreting results, the direction of these associations was changed.

^j^ Communicating with signs.

^k^ Clear speech, daytime engagement; for the purpose of correctly interpreting results, the direction of these associations was changed.

^l^ Communicating with writing, speech, gestures, sounds and pictures.

^m^ Compared to not living with parents.

## 3 RESULTS

### 3.1 Search and inclusion results

**FIGURE 1:**
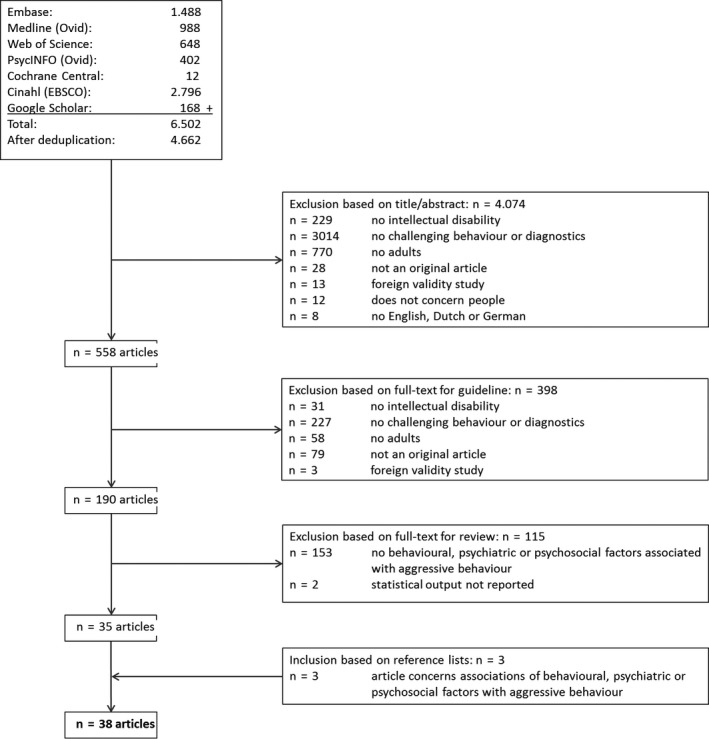
Flowchart of study inclusion

After removing duplicates, 4,662 publications were initially screened based on title and abstract and 588 publications were included for full‐text screening. Of those studies, 190 studies were included for the guideline development. Thirty‐five were included in the current review, and three additional articles were included from the reference list search, leading to a total of 38 studies being included in the review (Figure 1).

### 3.2 Characteristics of the 38 included publications

A full summary of all included publications can be found in Table 1. Included studies were carried out in the UK (*n* = 13), the USA (*n* = 13), Canada (*n* = 4), the Netherlands (*n* = 3), Australia (*n* = 1), Germany (*n* = 1), Hungary (*n* = 1), Norway (*n* = 1) and Sweden (*n* = 1).

Sixteen studies included people with all levels of intellectual disability (Bowring et al., 2017; Crocker et al., 2006; Hartley & MacLean, 2007; Hemmings et al., 2006; Horovitz et al., 2013; Hurley, 2008; Lundqvist, 2013; Matson & Rivet, 2008; Nøttestad & Linaker, 2002; Rojahn et al., 2004, 2010; Sappok et al., 2014; Thorson et al., 2008; Tsiouris et al., 2011; Turygin et al., 2013; Tyrer et al., 2006), six studies included people with mild intellectual disability (Alexander et al., 2010, 2015; Didden et al., 2009; Drieschner et al., 2013; Phillips & Rose, 2010; Tenneij et al., 2009), five studies included people with mild or moderate intellectual disability (Clark et al., 2016; Crocker et al., 2014; Davies et al., 2015; Larson et al., 2011; Lunsky et al., 2012), one study included people with mild, moderate or severe intellectual disability (Esbensen & Benson, 2006), one included people with moderate or severe intellectual disability (Totsika et al., 2008), one study included people with moderate, severe, or profound intellectual disability (Bernstein et al., 2015) and three included people with severe or profound intellectual disability (Cervantes & Matson, 2015; Matson et al., 2009; Ross & Oliver, 2002). Five studies did not report the level of intellectual disability for included people (Allen et al., 2012; Koritsas & Iacono, 2015; Lindsay et al., 2013; Novaco & Taylor, 2004; Owen et al., 2004).

Thirteen studies recruited people living in a residential facility (Bernstein et al., 2015; Cervantes & Matson, 2015; Drieschner et al., 2013; Horovitz et al., 2013; Matson et al., 2009; Matson & Rivet, 2008; Owen et al., 2004; Phillips & Rose, 2010; Rojahn et al., 2004, 2010; Thorson et al., 2008; Totsika et al., 2008; Turygin et al., 2013), 13 had a mix of settings (Allen et al., 2012; Bowring et al., 2017; Clark et al., 2016; Crocker et al., 2006; Esbensen & Benson, 2006; Hartley & MacLean, 2007; Hemmings et al., 2006; Koritsas & Iacono, 2015; Lundqvist, 2013; Lunsky et al., 2012; Nøttestad & Linaker, 2002; Sappok et al., 2014; Tyrer et al., 2006), six concerned people living in a forensic or inpatient treatment facility (Alexander et al., 2010, 2015; Didden et al., 2009; Lindsay et al., 2013; Novaco & Taylor, 2004; Tenneij et al., 2009) and two studies recruited people living in a community setting (Crocker et al., 2014; Tsiouris et al., 2011). Four studies did not report the living arrangements of individuals (Davies et al., 2015; Hurley, 2008; Larson et al., 2011; Ross & Oliver, 2002).

The majority of studies used informant reports (*n* = 23) (Allen et al., 2012; Bernstein et al., 2015; Bowring et al., 2017; Cervantes & Matson, 2015; Crocker et al., 2006; Drieschner et al., 2013; Esbensen & Benson, 2006; Hartley & MacLean, 2007; Horovitz et al., 2013; Koritsas & Iacono, 2015; Lundqvist, 2013; Matson et al., 2009; Matson & Rivet, 2008; Nøttestad & Linaker, 2002; Owen et al., 2004; Phillips & Rose, 2010; Rojahn et al., 2004, 2010; Ross & Oliver, 2002; Tenneij et al., 2009; Thorson et al., 2008; Totsika et al., 2008; Turygin et al., 2013), whereas others used chart reviews to collect relevant information (*n* = 7) (Alexander et al., 2010, 2015; Didden et al., 2009; Hurley, 2008; Lindsay et al., 2013; Sappok et al., 2014; Tyrer et al., 2006).

Three studies used a combination of chart reviews and informant reports (Clark et al., 2016; Lunsky et al., 2012; Tsiouris et al., 2011), two used a combination of chart reviews, informant reports and self‐reports (Crocker et al., 2014; Novaco & Taylor, 2004), two used a combination of self‐reports and informant reports (Davies et al., 2015; Larson et al., 2011) and one used a combination of chart reviews and self‐reports (Hemmings et al., 2006).

The sample sizes ranged from *n* = 24 (Ross & Oliver, 2002) to *n* = 4,069 (Tsiouris et al., 2011). Sixteen studies were determined to be of high quality (Bernstein et al., 2015; Cervantes & Matson, 2015; Clark et al., 2016; Crocker et al., 2014; Davies et al., 2015; Esbensen & Benson, 2006; Hartley & MacLean, 2007; Hemmings et al., 2006; Horovitz et al., 2013; Koritsas & Iacono, 2015; Matson et al., 2009; Matson & Rivet, 2008; Novaco & Taylor, 2004; Sappok et al., 2014; Tenneij et al., 2009; Tsiouris et al., 2011), the remaining 22 studies were rated as low quality (Alexander et al., 2010, 2015; Allen et al., 2012; Bowring et al., 2017; Crocker et al., 2006; Didden et al., 2009; Drieschner et al., 2013; Hurley, 2008; Larson et al., 2011; Lindsay et al., 2013; Lundqvist, 2013; Lunsky et al., 2012; Nøttestad & Linaker, 2002; Owen et al., 2004; Phillips & Rose, 2010; Rojahn et al., 2004, 2010; Ross & Oliver, 2002; Thorson et al., 2008; Totsika et al., 2008; Turygin et al., 2013; Tyrer et al., 2006). A total of 27 different instruments were used to assess aggressive behaviour.

Below, the identified associations are first presented according to topography of aggressive behaviour. To focus on factors for which most evidence was found, only those associations reported a minimum of four times is discussed in the text. Subsequently, we present the overall associations found for behavioural, psychiatric and psychosocial factors. Full results can be found in Tables 2, 3, 4, 5, 6, 7.

### 3.3 Association of behavioural, psychiatric and psychosocial factors per topography of aggressive behaviour

#### 3.3.1 Physically aggressive behaviour

Fifteen studies reported associations of 10 different factors with physically aggressive behaviour (Table 2) (Alexander et al., 2010; Bernstein et al., 2015; Crocker et al., 2006, 2014; Drieschner et al., 2013; Hartley & MacLean, 2007; Lunsky et al., 2012; Nøttestad & Linaker, 2002; Novaco & Taylor, 2004; Phillips & Rose, 2010; Ross & Oliver, 2002; Sappok et al., 2014; Totsika et al., 2008; Tsiouris et al., 2011; Tyrer et al., 2006). The presence of physical aggression was based on information from informants, case files, the challenging behaviour interview (CBI), MOAS(+), inventory for client and agent planning (ICAP), Disability assessment Schedule for Behaviour problems (DAS‐B) and the Individual Schedule. Five associations were studied four or more times; the association of physicially aggressive behaviour with (a) self‐injurious behaviour, (b) mood disorders, (c) neurotic, stress‐related and somatoform disorders, (d) personality disorders and (e) disorders of psychosocial development. Of these, none showed unanimous results.

#### 3.3.2 Verbally aggressive behaviour

Eight studies reported associations of six different factors with verbally aggressive behaviour (Table 3) (Alexander et al., 2010; Crocker et al., 2006, 2014; Drieschner et al., 2013; Lindsay et al., 2013; Ross & Oliver, 2002; Sappok et al., 2014; Tsiouris et al., 2011). The presence of verbal aggression was based on information from case files, the MOAS(+) and the CBI. Only the association with neurotic, stress‐related and somatoform disorders was reported four or more times, but this did not result in a unanimous conclusion.

#### 3.3.3 Destructive behaviour

Fourteen studies reported associations of eight different factors with destructive behaviour (Table 4) (Alexander et al., 2010, 2015; Allen et al., 2012; Crocker et al., 2006, 2014; Drieschner et al., 2013; Hartley & MacLean, 2007; Hemmings et al., 2006; Lindsay et al., 2013; Lunsky et al., 2012; Nøttestad & Linaker, 2002; Ross & Oliver, 2002; Sappok et al., 2014; Tsiouris et al., 2011). The presence of destructive behaviour was based on information from informants, case files, the Individual Schedule, MOAS(+), ICAP, disability assessment schedule (DAS) and the CBI. Six associations were studied four or more times; the association of destructive behaviour with (a) physically aggressive behaviour, (b) criminal behaviour, (c) schizophrenia and delusional disorders, (d) mood disorders, (e) personality disorders and (f) disorders of psychological development. None showed unanimous results.

#### 3.3.4 Sexually aggressive behaviour

Six studies reported associations of four different factors with sexually aggressive behaviour (Table 5) (Alexander et al., 2010; Cervantes & Matson, 2015; Crocker et al., 2006, 2014; Drieschner et al., 2013; Lindsay et al., 2013). The presence of sexual aggression was based on information from case files, the MOAS(+) and the diagnostic assessment of the severely handicapped (DASH‐II). No associations were studied four or more times.

#### 3.3.5 Self‐injurious behaviour

Twenty‐five studies reported associations of nine different factors with self‐injurious behaviour (Table 6) (Allen et al., 2012; Bernstein et al., 2015; Bowring et al., 2017; Clark et al., 2016; Crocker et al., 2006; Drieschner et al., 2013; Hemmings et al., 2006; Horovitz et al., 2013; Hurley, 2008; Larson et al., 2011; Lundqvist, 2013; Lunsky et al., 2012; Matson et al., 2009; Matson & Rivet, 2008; Nøttestad & Linaker, 2002; Owen et al., 2004; Rojahn et al., 2004, 2010; Ross & Oliver, 2002; Sappok et al., 2014; Tenneij et al., 2009; Thorson et al., 2008; Totsika et al., 2008; Tsiouris et al., 2011; Turygin et al., 2013). The presence of self‐injurious behaviour was based on information from case files, informant reports, self‐made questionnaires, the Individual Schedule, CBI, behaviour problems inventory (BPI)(‐S, ‐01), DASH‐II, MOAS(+), DAS, autism spectrum disorder ‐behaviour problems for adults (ASD‐BPA) and the staff observation and aggression scale‐revised (SOAS‐R). Twelve associations were studied four or more times. Of these, only one showed unanimous results and indicated a positive association of self‐injurious behaviour with aggression in general. The other eleven factors—physically aggressive behaviour, schizophrenia and delusional disorders, mood disorders, neurotic, stress‐related and somatoform disorders, personality disorders, disorders of psychological development, symptoms of schizophrenia and delusional disorders, symptoms of mood disorders, symptoms of behavioural syndromes associated with physi(ologi)cal factors, symptoms of disorders of psychological development and positive social skills—did not find unanimous results.

#### 3.3.6 Aggressive behaviour in general

Twenty‐one studies reported associations of eight different factors with aggressive behaviour in general (Table 7) (Allen et al., 2012; Bernstein et al., 2015; Bowring et al., 2017; Clark et al., 2016; Crocker et al., 2006; Davies et al., 2015; Didden et al., 2009; Drieschner et al., 2013; Esbensen & Benson, 2006; Hemmings et al., 2006; Horovitz et al., 2013; Koritsas & Iacono, 2015; Larson et al., 2011; Lundqvist, 2013; Matson & Rivet, 2008; Owen et al., 2004; Rojahn et al., 2004, 2010; Sappok et al., 2014; Tenneij et al., 2009; Thorson et al., 2008). The presence of aggression was based on information from informants, case files, self‐made questionnaires, the Individual Schedule, BPI(‐S, ‐01), MOAS(+), checklist of challenging behaviour (CCB), adult behaviour checklist (ABCL), scales of independent behaviour‐revised (SIB‐R), DAS, ASD‐BPA, Interview Protocol, ICAP and the SOAS‐R. Eleven associations were studied four times or more. Of these, one showed unanimous results, indicating a positive association of aggression in general with self‐injurious behaviour. Both the absence and the presence of an association were reported for disorders of psychological development, symptoms of schizophrenia and delusional disorders, symptoms of mood disorders, symptoms of neurotic, stress‐related and somatoform disorders, symptoms of disorders of psychological development, total psychiatric symptoms, aspecific psychiatric symptoms, communications skills, life events and positive social skills.

### 3.4 The overall associations of behavioural, psychiatric and psychosocial factors with aggressive behaviour

#### 3.4.1 Behavioural factors

The associations between various topographies of aggressive behaviours with behavioural factors have been reported in 11 studies (Alexander et al., 2015; Bernstein et al., 2015; Bowring et al., 2017; Clark et al., 2016; Crocker et al., 2006; Drieschner et al., 2013; Koritsas & Iacono, 2015; Lunsky et al., 2012; Nøttestad & Linaker, 2002; Rojahn et al., 2004; Tenneij et al., 2009). The majority of these studies found positive associations between the different topographies of aggressive behaviour, indicating a co‐occurrence of these topographies.

#### 3.4.2 Psychiatric factors

The associations between psychiatric factors and aggressive behaviour have been reported in 29 studies (Alexander et al., 2010, 2015; Allen et al., 2012; Bernstein et al., 2015; Bowring et al., 2017; Cervantes & Matson, 2015; Clark et al., 2016; Crocker et al., 2014; Davies et al., 2015; Didden et al., 2009; Drieschner et al., 2013; Hemmings et al., 2006; Horovitz et al., 2013; Hurley, 2008; Koritsas & Iacono, 2015; Larson et al., 2011; Lindsay et al., 2013; Lundqvist, 2013; Lunsky et al., 2012; Matson & Rivet, 2008; Rojahn et al., 2004, 2010; Ross & Oliver, 2002; Sappok et al., 2014; Thorson et al., 2008; Totsika et al., 2008; Tsiouris et al., 2011; Turygin et al., 2013; Tyrer et al., 2006). Some studies found an association of specific psychiatric disorders or symptoms with aggressive behaviours, whereas others reported the absence of the same associations. Hence, the results cannot clearly confirm nor exclude the presence of specific associations.

#### 3.4.3 Psychosocial factors

The association of aggressive behaviour with psychosocial factors has been reported in 16 studies (Alexander et al., 2015; Bowring et al., 2017; Clark et al., 2016; Crocker et al., 2006; Esbensen & Benson, 2006; Hartley & MacLean, 2007; Hemmings et al., 2006; Koritsas & Iacono, 2015; Lundqvist, 2013; Lunsky et al., 2012; Matson et al., 2009; Novaco & Taylor, 2004; Owen et al., 2004; Phillips & Rose, 2010; Sappok et al., 2014; Tyrer et al., 2006). Due to the low number of studies and conflicting outcomes, no clear results were found on the presence or absence of specific associations.

## 4 DISCUSSION

This study gives a comprehensive overview of the evidence concerning the associations of behavioural, psychiatric and psychosocial factors with different topographies of aggressive behaviour.

### 4.1 Topographies of aggression

Self‐injurious behaviour was the most studied type of aggression, followed by aggression in general. Physical aggression and destructive behaviour were studied fewer times, while studies including verbal and sexual aggression were scarce. This is surprising, as verbal aggression has been reported as the most common form of aggression in people with intellectual disability (Crocker et al., 2006; Drieschner et al., 2013; Tsiouris et al., 2011). Moreover, it has been noted that staff members experience the most impact of physical aggression and less so of self‐injurious behaviour, as they believe they have little control over this (Dilworth et al., 2011; Hensel et al., 2014). More research into the associations of behavioural, psychiatric and psychosocial factors with physical and verbal aggression is therefore advised.

### 4.2 Behavioural factors

Different topographies of aggressive behaviour seem to be associated with each other. This is in line with previous research that found a co‐occurrence of different topographies of challenging behaviour (Emerson et al., 2001; Lowe et al., 2007). Possible explanations include the presence of a shared underlying problem such as impulsivity, irritability, or a psychiatric or somatic illness, or that the response of a carer to one topography may elicit another (Nøttestad & Linaker, 2002).

Although there was a fair amount of studies on self‐injurious behaviour, physically aggressive behaviour, destructive behaviour and general aggressive behaviour, less studies have examined verbally aggressive behaviour and sexually aggressive behaviour. Even though the topographies regularly co‐occur, resulting in a more complex situation, it is possible that the specific topographies have specific factors underlying the emergence or continuation of that behaviour. Additional studies are therefore needed to understand factors that may be associated with the specific topographies.

### 4.3 Psychiatric factors

Most included studies focused on associations with psychiatric disorders and symptoms. They show mixed results for the existence of an association of these factors with aggressive behaviour, which is in line with previous statements that the relation between aggressive behaviour and psychiatric disorders or symptoms is not yet fully understood (Allen, 2008; Thakker et al., 2012). An association may be explained by different mechanisms, of which several have previously been proposed. First, there may be a shared aetiology for the aggressive behaviour and the psychiatric symptoms or disorders. Secondly, the aggressive behaviour may occur secondary to the psychiatric disorder, with the psychiatric disorder contributing to the aggressive behaviour or as an atypical presentation. Thirdly, aggressive behaviour may be the result from side‐effects of the pharmacological treatment of the psychiatric disorder (Allen, 2008; Emerson, 2001; Royal College of Psychiatrists, British Psychological Society and Royal College of Speech and Language Therapists, 2007; Thakker et al., 2012).

We also found that 15 studies focused on psychiatric disorders (Alexander et al., 2010, 2015; Bowring et al., 2017; Cervantes & Matson, 2015; Crocker et al., 2014; Drieschner et al., 2013; Horovitz et al., 2013; Hurley, 2008; Lindsay et al., 2013; Lunsky et al., 2012; Sappok et al., 2014; Thorson et al., 2008; Totsika et al., 2008; Tsiouris et al., 2011; Tyrer et al., 2006) and 15 studies focused on psychiatric symptoms (Allen et al., 2012; Bernstein et al., 2015; Clark et al., 2016; Davies et al., 2015; Didden et al., 2009; Hemmings et al., 2006; Koritsas & Iacono, 2015; Larson et al., 2011; Lundqvist, 2013; Matson & Rivet, 2008; Rojahn et al., 2004, 2010; Ross & Oliver, 2002; Turygin et al., 2013; Tyrer et al., 2006). One study focused on both disorders and symptoms (Tyrer et al., 2006). To diagnose, a person with intellectual disability with a psychiatric disorder is not easy, especially with increasing levels of disability (Flynn et al., 2017; Peña‐Salazar et al., 2018). Difficulties may originate from communication difficulties, cognitive issues or atypical presentations of the disorder. Although it may be difficult to diagnose a person with a certain disorder, symptoms suggestive of such a disorder should not be considered similar to an actual diagnosis. Moreover, disorders and symptoms may have different associations with aggressive behaviour. We therefore decided to present results separately for psychiatric disorders and symptoms indicative of specific psychiatric disorders. In the current review, a disorder was classified as such when it was based on criteria outlined in the DSM or ICD and was made by a qualified professional. Surprisingly, none of the included studies described the use of the DC‐LD or DM‐ID for the diagnostic process, even though these instruments are designed specifically for people with intellectual disability. Based on the limited evidence included in the current review, it cannot be determined whether associations with aggressive behaviour are different for diagnosed disorders and the presence of symptoms.

### 4.4 Psychosocial factors

Psychosocial factors are not as widely studied as psychiatric factors. However, factors such as the quality of received care and quality and frequency of interpersonal interactions, both with caregivers and within the social network, have been deemed important in the prevention or emergence of aggressive behaviour (Beadle‐Brown et al., 2016; Bigby & Beadle‐Brown, 2018; Embregts et al., 2009; Rose, 2011). For instance, people with intellectual disability may present with aggressive behaviour to secure attention from caregivers (Lloyd & Kennedy, 2014), in an effort to increase the frequency of interactions. However, caregivers may respond less friendly when faced with aggressive behaviour (Willems et al., 2014), resulting in a lower quality of interactions. These examples underscore that psychosocial factors may have a complex and conflicting influence of the presence of aggression, warranting additional research.

The definition of intellectual disability in the DSM‐5 further underscores the importance of adaptive behaviour—including conceptual, social and practical skills—in the functioning of a person with intellectual disability (American Psychiatric Association, 2013). The functioning of a person with intellectual disability is described by the American Association on Intellectual and Developmental Disabilities (AAIDD) as a complex interaction of different domains, while taking the need for support into account (Schalock et al., 2010). It is assumed that when the demands placed upon a person do not match the abilities of that person, the resulting disbalance may lead to aggressive or self‐injurious behaviour (Bowring et al., 2017; Sappok et al., 2014; Totsika et al., 2008). As a result of the low number of included studies, the current review was unable to confirm or disprove this theory, which requires additional examinations.

## 5 LIMITATIONS AND FUTURE RESEARCH

The included studies were heterogeneous in terms of definitions, methodologies, study population and settings used. The large number of different methods used to assess aggressive behaviour and the different psychosocial factors that were studied might make it more difficult to compare results from different studies. In the 38 included studies, 27 different questionnaires, checklists or methodologies were used to determine aggressive behaviour. Definitions of aggressive behaviour varied widely, both on the severity of behaviour necessary to be labelled as such (for instance “any aggressive behaviour” to “frequent/and or severe behaviour”) and different time periods in which the behaviour had to occur (ranging from a week to a year, or to “a history of aggression in the case file”). Similarly, the different psychosocial factors were recorded using a range of different tools as described in Table 1. The study population was heterogeneous in that some studies only included people with mild intellectual disability, whereas others only included people with a severe or profound disability level. Most studies focused on people in residential settings, whereas others included all different forms of living arrangements or only included people in a forensic setting. A more consistent use of methodologies and definitions and a stratified analysis would be beneficial to compare studies among each other and to compile the data in a meta‐analysis.

Aggressive behaviour will oftentimes be the result of a combination of multiple factors or even of an interaction between different factors (Embregts et al., 2009; Koritsas & Iacono, 2015; Nøttestad & Linaker, 2002; Schalock et al., 2010). These factors may either contribute to the aggressive behaviour, or prevent its emergence. To understand the origin of the behaviour for a specific person, a multifactorial approach is necessary. The same holds true for the investigation of factors associated with aggressive behaviour. This would require multivariate analyses, including not only the factors in the current review but also biological factors and personal characteristics. Several of the included studies did apply multivariate analyses rather than univariate ones and one applied mediation analyses to further understand the associations (Table 1).

The methodological quality of the included studies varied greatly; the sample size ranged from 24 (Ross & Oliver, 2002) to 4,069 (Tsiouris et al., 2011) adults. As Drieschner et al. (2013) have stated, a small sample size may lead to over‐ or underestimation of an association since a large percentage of the aggressive incidents were due to a small group of people. Furthermore, most studies applied a cross‐sectional or retrospective approach. A prospective longitudinal study design may be more informative for directionality of the associations.

## 6 CONCLUSIONS AND CLINICAL IMPLICATIONS

The results of this review suggest that at the moment, there is no clarity on factors contributing to aggressive behaviour. Although a variety of behavioural, psychosocial and psychiatric factors have been studied in literature, none showed consistent, unanimous results. This supports the notion that the aetiology of aggressive behaviour is often specific to a certain person in a certain context. Aggression is often the result of multiple factors within the person and in the context and interactions between these factors (Ali et al., 2014; Antonacci et al., 2008). Some factors may contribute to the emergence of the behaviour, whereas others may be protective. It is therefore important that a functional assessment is performed on an individual basis (Embregts et al., 2009). This may require a multidisciplinary effort of for instance a physician to investigate possible somatic factors, a psychologist to investigate psychological factors or communication skills and a caregiver to investigate contextual factors or life events. Based on the outcomes of a functional assessment, an integrative hypothesis can be formed which may aid in the choice for the most appropriate (combination of) intervention(s) (Ali et al., 2014; Kerr et al., 2013). The information may guide treatment and future preventive efforts. This is in agreement with guidelines on challenging behaviour which state that a personalized intervention focused on causal factors of a behaviour is desirable over a general approach focused on the symptoms of the behaviour (Embregts et al., 2019; National Institute & for Health & Clinical Excellence, 2015). At the moment, there is not sufficient evidence to determine which factors are most likely to contribute to the emergence or continuation of aggressive behaviour, although the current review suggests that certain behavioural, psychiatric and psychosocial factors may contribute. In light of the much mentioned diagnostic overshadowing (Peña‐Salazar et al., 2018), it is recommended that such factors are part of the functional assessment. To better guide functional assessments, it is imperative that more research on factors contributing to aggressive behaviour is undertaken, which also focuses on understanding the causality of the associations.

## CONFLICT OF INTEREST

None of the authors has any potential conflict of interest related to this manuscript.

## APPENDIX 1 Search terms

***Embase.com (Embase incl. Medline): 1488***

**(**“mental deficiency”/**mj**/exp OR “mentally disabled person”/mj/de OR “intellectual impairment”/**mj/**de OR (((intellect* OR mental*) NEAR/4 (disab* OR impair* OR defici* OR retard* OR disfunct* OR handicap* OR incapacit*)) OR ((intellect*) NEAR/3 (disorder*)) OR ((cogniti*) NEXT/1 (retard*)) OR ((Down OR Hunter OR Hurler OR Sanfilippo) NEAR/3 (syndrome*)) *OR “fragile X” OR “happy puppet” OR “Prader Willi”* OR ((Leigh) NEXT/1 (disease*))):ti**) AND (**“behaviour disorder”/exp OR “aggression”/exp OR “antisocial behaviour”/exp OR “aversive behaviour”/exp OR “behavioural stress”/de OR “avoidance behaviour”/de OR “escape behaviour”/de OR “psychosocial withdrawal”/de OR “social avoidance behaviour”/de OR “drinking behaviour”/de OR “addiction”/exp OR “high risk behaviour”/de OR “malingering”/de OR “masochism”/de OR “mental dissociation”/de OR “misconduct”/exp OR “perseveration”/de OR “sadism”/de OR “sadomasochism”/de OR “sexual deviation”/de OR “stereotypy”/de OR “substance use”/exp OR “wandering behaviour”/de OR “antisocial behaviour”/exp OR “conflict”/de OR “anger”/exp OR “anhedonia”/exp OR “fear”/exp OR “frustration”/de OR “nervousness”/exp OR “enuresis”/exp OR “obsession”/exp OR “compulsion””/de OR “paranoia”/de *OR “crime”/exp OR prison/exp OR “prisoner”/de OR “detention”/de OR “offender”/de* OR (((challenging OR aberra* OR abnormal OR disturb* OR disorder* OR crisis OR crises OR devian* OR disrupt* OR problem* OR disapprov*) NEAR/3 (behavi* OR conduct OR communic* OR eat* OR drinking OR “impulse control”)) OR ((psychomotor* OR *psychosocial*) NEAR/3 (disorder* OR deficien* OR disturb* OR impairment* OR retard*)) OR elopemen* OR automutilat* OR ((self) NEAR/3 (injur* OR harm* OR mutilat* OR poison*)) OR mutism* OR mute OR aphonia* OR ((drug* OR alcohol OR tobacco OR substance*) NEAR/3 (seeking OR abus* OR consum* *OR use* OR using OR problem* OR depend*)) OR anorexia* OR bulimia* OR addict* OR “food aversion” OR ((antisocial OR “anti social” OR drinking OR dangerous* OR risk*) NEAR/3 (behav*)) *OR killing OR murder** OR pica OR geophagia* OR pagophagia* OR trichophagia* OR kleptomania* OR pyromania* OR trichotillomania* OR akines* OR akinet* OR bradykine* OR hypokine* OR bruxism* OR ((dent* OR tooth OR teeth) NEAR/3 (grind* OR clench*)) OR cataton* OR hyperacti* OR hypoacti* OR suicid* OR “acting out” OR avoid* OR escap* OR withdraw* OR dissociat* OR misconduct* OR perseverati* OR sadis* OR sadomasochis* OR cruel* OR (sexual NEAR/3 (devia*)) OR stereotyp* OR alcoholism OR cannabis OR tobacco OR smoking OR wandering OR crim* OR delinquen* OR neglect* OR negligen* OR stalk* OR violen* OR assault OR battering OR homocide* OR murder* OR assassinat* OR tortur* OR hostil* OR aggressi* OR anger* OR hate OR rage* OR bullying OR provocat* OR threat* OR tantrum OR conflict* OR anhedonia* OR fear* OR anxiet* OR anxious* OR frustrat* OR nervousness OR nervosit* OR enuresis OR “bed wetting” OR bedwetting OR coprophag* OR hoarding OR obsessi* *OR compuls** OR paranoi* OR escalati* *OR runaway* OR crime* OR criminal* OR prison* OR offender* OR jail OR delinquen**):ab,ti**) AND (**“adult”/exp OR (adult* OR grownup* OR "middle aged" *OR ((grown) NEXT/1 (up*))* OR elderly):ab,ti**) AND (**“social interaction”/de OR “interpersonal communication”/exp OR “family relation”/exp OR “friendship”/de OR “caregiver support”/de OR “acoustics”/de OR “environmental parameters”/exp OR “light related phenomena”/exp OR “temperature related phenomena”/exp OR “incidence”/exp OR “prevalence”/exp OR “etiology”/exp OR (((social OR interpersonal* OR caregiver* OR staff OR psychosocial* OR family) NEAR/3 (interact* OR function* OR relati* OR communicat* OR support*)) OR friend* OR ((verbal* OR nonverbal* OR skill*) NEAR/3 (communicat*)) OR acoustic* OR noise* OR sound* OR temperature* OR heat* OR cold* OR light* OR lumen* OR lumin* OR illuminat* OR photo* OR optic* OR incidence* OR prevalen* OR etiolog* OR aetiolog*):ab,ti**) NOT** ([animals]/lim NOT [humans]/lim) **NOT (**“Conference Abstract”/it OR “Editorial”/it**) AND** [2002‐2017]/py

***Medline (Ovid): 988***

**(***"Mentally Disabled People"/ OR exp *"Intellectual Disability"/ OR (((intellect* OR mental*) ADJ6 (disab* OR impair* OR defici* OR retard* OR disfunct* OR handicap* OR incapacit*)) OR ((intellect*) ADJ3 (disorder*)) OR ((cogniti*) ADJ1 (retard*)) OR ((Down OR Hunter OR Hurler OR Sanfilippo ) ADJ3 (syndrome*)) *OR "fragile X" OR "happy puppet" OR "Prader Willi"* OR ((Leigh) ADJ (disease*))).ti.**) AND (**exp "Social Behaviour Disorders"/ OR exp "Aggression"/ OR "Problem Behaviour"/ OR "Hate"/ OR "Runaway Behaviour"/ OR exp "Drinking Behaviour"/ OR "Alcoholism"/ OR exp "Feeding and Eating Disorders"/ OR "Malingering"/ OR "Masochism"/ OR "Sadism"/ OR exp "Self‐Injurious Behaviour"/ OR "Wandering Behaviour"/ OR "Conflict (Psychology)"/ OR exp "Anger"/ OR "Anhedonia"/ OR exp "Fear"/ OR "Frustration"/ OR exp "Enuresis"/ OR exp "Obsessive Behaviour"/ OR exp "Compulsive Behaviour"/ *OR "Paranoid Behaviour"/ OR exp "Crime"/ OR exp "Criminal Psychology"/ OR "Criminal Behaviour"/ OR "Dangerous Behaviour"/ OR "Prisons"/ OR "Prisoners"/ OR "Criminals"/* OR (((challenging OR aberra* OR abnormal OR disturb* OR disorder* OR crisis OR crises OR devian* OR disrupt* OR problem* OR disapprov* OR binge) ADJ3 (behavi* OR conduct OR communic* OR eat* OR drinking OR "impulse control")) OR ((psychomotor* *OR psychosocial*) ADJ3 (disorder* OR deficien* OR disturb* OR impairment* OR retard*)) OR elopemen* OR automutilat* OR ((self) ADJ3 (injur* OR harm* OR mutilat* OR poison*)) OR mutism* OR mute OR aphonia* OR ((drug* OR alcohol OR tobacco OR substance*) ADJ3 (seeking OR abus* OR consum* OR using OR problem* OR depend*)) OR anorexia* OR bulimia* OR addict* OR "food aversion" OR ((antisocial OR "anti social" OR *drinking OR dangerous* OR risk**) ADJ3 (behav*)) *OR killing OR murder** OR pica OR geophagia* OR pagophagia* OR trichophagia* OR kleptomania* OR pyromania* OR trichotillomania* OR akines* OR akinet* OR bradykine* OR hypokine* OR bruxism* OR ((dent* OR tooth OR teeth) ADJ3 (grind* OR clench*)) OR cataton* OR hyperacti* OR hypoacti* OR suicid* OR "acting out" OR avoid* OR escap* OR withdraw* OR dissociat* OR misconduct* OR perseverati* OR sadis* OR sadomasochis* OR cruel* OR (sexual ADJ3 (devia*)) OR stereotyp* OR alcoholism OR cannabis OR tobacco OR smoking OR wandering OR crim* OR delinquen* OR neglect* OR negligen* OR stalk* OR violen* OR assault OR battering OR homocide* OR murder* OR assassinat* OR tortur* OR hostil* OR aggressi* OR anger* OR hate OR rage OR bullying OR provocat* OR threat* OR tantrum OR conflict* OR anhedonia* OR fear* OR anxiet* OR anxious* OR frustrat* OR nervousness OR nervosit* OR enuresis OR "bed wetting" OR bedwetting OR coprophag* OR hoarding OR obsessi** OR compuls** OR paranoi* OR escalati* *OR runaway* OR crime* OR criminal* OR prison* OR offender* OR jail OR delinquen**).ab,ti.**) AND (**exp "Adult"/ OR (adult* OR grownup* *OR ((grown) ADJ (up*))* OR "middle aged" OR elderly).ab,ti.**) AND (**exp "Interpersonal Relations"/ OR "Friends"/ OR exp "Family Relations"/ OR "Communication"/ OR "Acoustics"/ OR "Environment"/ OR "Noise"/ OR exp "Light"/ OR exp "Temperature"/ OR "Incidence"/ OR "Prevalence"/ OR *etiology.hw*. OR (((social OR interpersonal* OR caregiver* OR staff OR psychosocial*OR family) ADJ3 (interact* OR function* OR relati* OR communicat* OR support*)) OR friend* OR ((verbal* OR nonverbal* OR skill*) ADJ3 (communicat*)) OR acoustic* OR noise* OR sound* OR temperature* OR heat* OR cold* OR light* OR lumen* OR lumin* OR illuminat* OR photo* OR optic* OR incidence* OR prevalen* OR etiolog* OR aetiolog*).ab,ti.**) NOT (**exp animals/ NOT humans/**)** **NOT (**congresses OR editorial**)**.pt.

*Limit to yr="2002‐2017"*

***Cochrane Central (trials): 12*** *(no publication year)*

**(**(((intellect* OR mental*) *NEAR/4* (disab* OR impair* OR defici* OR retard* OR disfunct* OR handicap* OR incapacit*)) OR ((intellect*) NEAR/3 (disorder*)) OR ((cogniti*) NEXT/1 (retard*)) OR ((Down OR Hunter OR Hurler OR Sanfilippo OR “fragile X” OR “happy puppet” OR “Prader Willi”) NEAR/3 (syndrome*)) OR ((Leigh) NEXT/1 (disease*))):ti**) AND (**(((challenging OR aberra* OR abnormal OR disturb* OR disorder* OR crisis OR crises OR devian* OR disrupt* OR problem* OR disapprov*) NEAR/3 (behavi* OR conduct OR communic* OR eat* OR drinking OR “impulse control”)) OR ((psychomotor* OR *psychosocial*) NEAR/3 (disorder* OR deficien* OR disturb* OR impairment* OR retard*)) OR elopemen* OR automutilat* OR ((self) NEAR/3 (injur* OR harm* OR mutilat* OR poison*)) OR mutism* OR mute OR aphonia* OR ((drug* OR alcohol OR tobacco OR substance*) NEAR/3 (seeking OR abus* OR consum* *OR use* OR using OR problem* OR depend*)) OR anorexia* OR bulimia* OR addict* OR “food aversion” OR ((antisocial OR “anti social” OR drinking OR dangerous* OR risk*) NEAR/3 (behav*)) *OR killing OR murder** OR pica OR geophagia* OR pagophagia* OR trichophagia* OR kleptomania* OR pyromania* OR trichotillomania* OR akines* OR akinet* OR bradykine* OR hypokine* OR bruxism* OR ((dent* OR tooth OR teeth) NEAR/3 (grind* OR clench*)) OR cataton* OR hyperacti* OR hypoacti* OR suicid* OR “acting out” OR avoid* OR escap* OR withdraw* OR dissociat* OR misconduct* OR perseverati* OR sadis* OR sadomasochis* OR cruel* OR (sexual NEAR/3 (devia*)) OR stereotyp* OR alcoholism OR cannabis OR tobacco OR smoking OR wandering OR crim* OR delinquen* OR neglect* OR negligen* OR stalk* OR violen* OR assault OR battering OR homocide* OR murder* OR assassinat* OR tortur* OR hostil* OR aggressi* OR anger* OR hate OR rage* OR bullying OR provocat* OR threat* OR tantrum OR conflict* OR anhedonia* OR fear* OR anxiet* OR anxious* OR frustrat* OR nervousness OR nervosit* OR enuresis OR “bed wetting” OR bedwetting OR coprophag* OR hoarding OR obsessi* *OR compuls** OR paranoi* OR escalati* *OR runaway* OR crime* OR criminal* OR prison* OR offender* OR jail OR delinquen**):ab,ti**) AND (**(adult* OR grownup* OR "middle aged" *OR ((grown) NEXT/1 (up*))* OR elderly):ab,ti**) AND** ((((social OR interpersonal* OR caregiver* OR staff OR psychosocial* OR family) NEAR/3 (interact* OR function* OR relati* OR communicat* OR support*)) OR friend* OR ((verbal* OR nonverbal* OR skill*) NEAR/3 (communicat*)) OR acoustic* OR noise* OR sound* OR temperature* OR heat* OR cold* OR light* OR lumen* OR lumin* OR illuminat* OR photo* OR optic* OR incidence* OR prevalen* OR etiolog* OR aetiolog*):ab,ti)

***PsycInfo (Ovid): 402***

**(**exp *"Intellectual Development Disorder"/ OR (((intellect* OR mental*) ADJ4 (disab* OR impair* OR defici* OR retard* OR disfunct* OR handicap* OR incapacit*)) OR ((intellect*) ADJ3 (disorder*)) OR ((cogniti*) ADJ1 (retard*)) OR ((Down OR Hunter OR Hurler OR Sanfilippo OR "fragile X" OR "happy puppet" OR "Prader Willi") ADJ3 (syndrome*)) OR ((Leigh) ADJ (disease*))).ti.**) AND (** *exp "Behaviour Disorders"/* OR *exp "Aggressive Behaviour"/* OR *exp "Antisocial Behaviour"/ OR exp "Crime"/ OR "Forensic Psychology"/ OR exp "Aversion"/* OR *exp "Alcohol Drinking Patterns"/ OR exp "Eating Disorders"/ OR "Binge Eating"/ OR "Malingering"/ OR exp "Sadomasochism"/* OR *exp "Self‐Destructive Behaviour"/ OR "Wandering Behaviour"/* *OR exp "Anger"/ OR "Anhedonia"/* OR *exp "Fear"/ OR exp "Anxiety"/ OR "Frustration"/ OR "Urinary Incontinence"/* *OR exp "Obsessions"/* OR *exp "Compulsions"/ OR exp "Hoarding Behaviour"/ OR "Paranoia"/* OR (((challenging OR aberra* OR abnormal OR disturb* OR disorder* OR crisis OR crises OR devian* OR disrupt* OR problem* OR disapprov* OR binge*) ADJ3 (behavi* OR conduct OR communic* OR eat* OR drinking OR "impulse control")) OR ((psychomotor* OR *psychosocial*) ADJ3 (disorder* OR deficien* OR disturb* OR impairment* OR retard*)) OR elopemen* OR automutilat* OR ((self) ADJ3 (injur* OR harm* OR mutilat* OR poison*)) OR mutism* OR mute OR aphonia* OR ((drug* OR alcohol OR tobacco OR substance*) ADJ3 (seeking OR abus* OR consum* OR using OR problem* OR depend*)) OR anorexia* OR bulimia* OR addict* OR "food aversion" OR ((antisocial OR "anti social" *OR drinking OR dangerous* OR risk**) ADJ3 (behav*)) *OR killing OR murder** OR pica OR geophagia* OR pagophagia* OR trichophagia* OR kleptomania* OR pyromania* OR trichotillomania* OR akines* OR akinet* OR bradykine* OR hypokine* OR bruxism* OR ((dent* OR tooth OR teeth) ADJ3 (grind* OR clench*)) OR cataton* OR hyperacti* OR hypoacti* OR suicid* OR "acting out" OR avoid* OR escap* OR withdraw* OR dissociat* OR misconduct* OR perseverati* OR sadis* OR sadomasochis* OR cruel* OR (sexual ADJ3 (devia*)) OR stereotyp* OR alcoholism OR cannabis OR tobacco OR smoking OR wandering OR crim* OR delinquen* OR neglect* OR negligen* OR stalk* OR violen* OR assault OR battering OR homocide* OR murder* OR assassinat* OR tortur* OR hostil* OR aggressi* OR anger* OR hate OR rage OR bullying OR provocat* OR threat* OR tantrum OR conflict* OR anhedonia* OR fear* OR anxiet* OR anxious* OR frustrat* OR nervousness OR nervosit* OR enuresis OR "bed wetting" OR bedwetting OR coprophag* OR hoarding OR obsessi* *OR compuls** OR paranoi* OR escalati* *OR runaway* OR crime* OR criminal* OR prison* OR offender* OR jail OR delinquen**).ab,ti.**) AND (**"adult"/ OR (adult* OR grownup* *OR ((grown) ADJ (up*))* OR "middle aged" OR elderly).ab,ti.**) AND (** *exp "Interpersonal Communication"/ OR "Friendship"/ OR "Family Relations"/* *OR "Communication"/ OR "Acoustics"/ OR "Environmental Effects"/* *OR "Noise Effects"/ OR exp "Illumination"/* *OR exp "Temperature Effects"/* *OR "Epidemiology"/ OR "Etiology"/* OR (((social OR interpersonal* OR caregiver* OR staff OR psychosocial*OR family) ADJ3 (interact* OR function* OR relati* OR communicat* OR support*)) OR friend* OR ((verbal* OR nonverbal* OR skill*) ADJ3 (communicat*)) OR acoustic* OR noise* OR sound* OR temperature* OR heat* OR cold* OR light* OR lumen* OR lumin* OR illuminat* OR photo* OR optic* OR incidence* OR prevalen* OR etiolog* OR aetiolog*).ab,ti.**)**

*Limit to yr="2002‐2017"*

***Web of Science: 648***

**TI=(**((intellect* OR mental*) NEAR/4 (disab* OR impair* OR defici* OR retard* OR disfunct* OR handicap* OR incapacit*)) OR ((intellect*) NEAR/2 (disorder*)) OR ((cogniti*) NEAR/1 (retard*)) OR ((Down OR Hunter OR Hurler OR Sanfilippo OR "fragile X" OR "happy puppet" OR "Prader Willi") NEAR/2 (syndrome*)) OR ((Leigh) NEAR/1 (disease*))**) AND TS=((**(((challenging OR aberra* OR abnormal OR disturb* OR disorder* OR crisis OR crises OR devian* OR disrupt* OR problem* OR compulsive OR disapprov*) NEAR/2 (behavi* OR conduct OR communic* OR eat* OR drinking OR "impulse control")) OR ((psychomotor* OR *psychosocial*) NEAR/2 (disorder* OR deficien* OR disturb* OR impairment* OR retard*)) OR elopemen* OR automutilat* OR ((self) NEAR/2 (injur* OR harm* OR mutilat* OR poison*)) OR mutism* OR mute OR aphonia* OR ((drug* OR alcohol OR tobacco OR substance*) NEAR/2 (seeking OR abus* OR consum* OR using OR problem* OR depend*)) OR anorexia* OR bulimia* OR addict* OR "food aversion" OR ((antisocial OR "anti social" OR drinking OR dangerous*) NEAR/2 (behav*)) *OR killing OR murder** OR pica OR geophagia* OR pagophagia* OR trichophagia* OR kleptomania* OR pyromania* OR trichotillomania* OR akines* OR akinet* OR bradykine* OR hypokine* OR bruxism* OR ((dent* OR tooth OR teeth) NEAR/2 (grind* OR clench*)) OR cataton* OR hyperacti* OR hypoacti* OR suicid* OR "acting out" OR avoid* OR escap* OR withdraw* OR dissociat* OR misconduct* OR perseverati* OR sadis* OR sadomasochis* OR cruel* OR (sexual NEAR/2 (devia*)) OR stereotyp* OR alcoholism OR cannabis OR tobacco OR smoking OR wandering OR crim* OR delinquen* OR neglect* OR negligen* OR stalk* OR violen* OR assault OR battering OR homocide* OR murder* OR assassinat* OR tortur* OR hostil* OR aggressi* OR anger* OR hate OR rage* OR bullying OR provocat* OR threat* OR tantrum OR conflict* OR anhedonia* OR fear* OR anxiet* OR anxious* OR frustrat* OR nervousness OR nervosit* OR enuresis OR "bed wetting" OR bedwetting OR coprophag* OR hoarding OR obsessi* OR compuls* OR paranoi* OR escalati* *OR runaway* OR crime* OR criminal* OR prison* OR offender* OR jail OR delinquen**)**) AND (**(adult* OR grownup* *OR "grown up" OR "grown ups"* OR "middle aged" OR elderly)**) AND (**(((social OR interpersonal* OR caregiver* OR staff OR psychosocial* OR family) NEAR/2 (interact* OR function* OR relati* OR communicat* OR support*)) OR friend* OR ((verbal* OR nonverbal* OR skill*) NEAR/2 (communicat*)) OR acoustic* OR noise* OR sound* OR temperature* OR heat* OR cold* OR light* OR lumen* OR lumin* OR illuminat* OR photo* OR optic* OR incidence* OR prevalen* OR etiolog* OR aetiolog*)**))** AND DT=(Article) AND LA=(English)

*Timespan=2002‐2017*

***Cinahl (EBSCO): 2796***

**(** *MJ "Mentally Disabled People+" OR MJ "Intellectual Disability+"* OR (((intellect* OR mental*) N5 (disab* OR impair* OR defici* OR retard* OR disfunct* OR handicap* OR incapacit*)) OR ((intellect*) N2 (disorder*)) OR ((cogniti*) N1 (retard*)) OR ((Down OR Hunter OR Hurler OR Sanfilippo ) N2 (syndrome*)) OR "fragile X" OR "happy puppet" OR "Prader Willi" OR ((Leigh) N1 (disease*))).ti.**) AND (** *MH "Social Behaviour Disorders+" OR MH "Drinking Behaviour+"* OR*MH "Substance Abuse+"* *OR MH "Eating Disorders+" OR "OR MH "Risk Taking Behaviour+"* *OR MH "Conflict (Psychology)+" OR MH "Anxiety+"* *OR MH "Fear+"* *OR MH "Enuresis+"* *OR MH "Obsessive‐Compulsive Disorder+" OR MH "Crime+" OR MH "Criminal Psychology+" OR MH "Public Offenders+"* OR (((challenging OR aberra* OR abnormal OR disturb* OR disorder* OR crisis OR crises OR devian* OR disrupt* OR problem* OR disapprov* OR binge) N2 (behavi* OR conduct OR communic* OR eat* OR drinking OR "impulse control")) OR ((psychomotor* *OR psychosocial*) N2 (disorder* OR deficien* OR disturb* OR impairment* OR retard*)) OR elopemen* OR automutilat* OR ((self) N2 (injur* OR harm* OR mutilat* OR poison*)) OR mutism* OR mute OR aphonia* OR ((drug* OR alcohol OR tobacco OR substance*) N2 (seeking OR abus* OR consum* OR using OR problem* OR depend*)) OR anorexia* OR bulimia* OR addict* OR "food aversion" OR ((antisocial OR "anti social" OR *drinking OR dangerous* OR risk**) N2 (behav*)) *OR killing OR murder** OR pica OR geophagia* OR pagophagia* OR trichophagia* OR kleptomania* OR pyromania* OR trichotillomania* OR akines* OR akinet* OR bradykine* OR hypokine* OR bruxism* OR ((dent* OR tooth OR teeth) N2 (grind* OR clench*)) OR cataton* OR hyperacti* OR hypoacti* OR suicid* OR "acting out" OR avoid* OR escap* OR withdraw* OR dissociat* OR misconduct* OR perseverati* OR sadis* OR sadomasochis* OR cruel* OR (sexual N2 (devia*)) OR stereotyp* OR alcoholism OR cannabis OR tobacco OR smoking OR wandering OR crim* OR delinquen* OR neglect* OR negligen* OR stalk* OR violen* OR assault OR battering OR homocide* OR murder* OR assassinat* OR tortur* OR hostil* OR aggressi* OR anger* OR hate OR rage OR bullying OR provocat* OR threat* OR tantrum OR conflict* OR anhedonia* OR fear* OR anxiet* OR anxious* OR frustrat* OR nervousness OR nervosit* OR enuresis OR "bed wetting" OR bedwetting OR coprophag* OR hoarding OR obsessi* *OR compuls** OR paranoi* OR escalati* *OR runaway* OR crime* OR criminal* OR prison* OR offender* OR jail OR delinquen**)**) AND (** *MH "Adult+"* OR (adult* OR grownup* OR "middle aged" OR elderly)**) AND (**MH "Interpersonal Relations+" OR MH "Communication+" OR MH "Acoustics+" OR MH "Environment+" OR MH "Optics+" OR (((social OR interpersonal* OR caregiver* OR staff OR psychosocial*OR family) N2 (interact* OR function* OR relati* OR communicat* OR support*)) OR friend* OR ((verbal* OR nonverbal* OR skill*) N2 (communicat*)) OR acoustic* OR noise* OR sound* OR temperature* OR heat* OR cold* OR light* OR lumen* OR lumin* OR illuminat* OR photo* OR optic* OR incidence* OR prevalen* OR etiolog* OR aetiolog*)**)**

*Limiters: Published Date: 20020101‐20171231*

***Google Scholar: 200*** *(top relevance, no publication year)*

**"**intellectual disabled|impaired|disability**"** "challenging behaviour"|addiction|criminal|suicidal|"eating disorder"|violence|antisocial adult interpersonal|caregiver|communication|noise|sounds|optics|light|temperature|heat|cold|incidence|etiology|prevalence

## References

[jar12809-bib-0001] Alexander, R. T. , Chester, V. , Green, F. N. , Gunaratna, I. , & Hoare, S. (2015). Arson or fire setting in offenders with intellectual disability: Clinical characteristics, forensic histories, and treatment outcomes. Journal of Intellectual & Developmental Disability, 40(2), 189–197. 10.3109/13668250.2014.998182

[jar12809-bib-0002] Alexander, R. T. , Green, F. N. , O'Mahony, B. , Gunaratna, I. J. , Gangadharan, S. K. , & Hoare, S. (2010). Personality disorders in offenders with intellectual disability: A comparison of clinical, forensic and outcome variables and implications for service provision. Journal of Intellectual Disability Research, 54(7), 650–658. 10.1111/j.1365-2788.2010.01248.x 20136682

[jar12809-bib-0003] Ali, A. , Blickwedel, J. , & Hassiotis, A. (2014). Interventions for challenging behavior in intellectual disability. Advances in Mental Health and Intellectual Disabilities, 20, 184–192.

[jar12809-bib-0004] Allen, D. (2008). The relationship between challenging behaviour and mental ill‐health in people with intellectual disabilities: A review of current theories and evidence. Journal of Intellectual Disabilities, 12(4), 267–294. 10.1177/1744629508100494 19074934

[jar12809-bib-0005] Allen, D. , Lowe, K. , Matthews, H. , & Anness, V. (2012). Screening for psychiatric disorders in a total population of adults with intellectual disability and challenging behaviour using the PAS‐ADD checklist. Journal of Applied Research in Intellectual Disabilities, 25(4), 342–349. 10.1111/j.1468-3148.2011.00670.x 22711482

[jar12809-bib-0006] American Psychiatric Association (2013). Diagnostic and statistical manual of mental disorders (DSM‐5^®^). American Psychiatric Publishing.

[jar12809-bib-0007] Antonacci, D. J. , Manuel, C. , & Davis, E. (2008). Diagnosis and treatment of aggression in individuals with developmental disabilities. The Psychiatric Quarterly, 79, 225–247. 10.1007/s11126-008-9080-4 18726157

[jar12809-bib-0008] Beadle‐Brown, J. , Leigh, J. , Whelton, B. , Richardson, L. , Beecham, J. , Baumker, T. , & Bradshaw, J. (2016). Quality of life and quality of support for people with severe intellectual disability and complex needs. Journal of Applied Research in Intellectual Disabilities, 29(5), 409–421. 10.1111/jar.12200 25998790

[jar12809-bib-0009] Bernstein, A. M. , Visconti, K. J. , Csorba, J. , Radvanyi, K. , & Rojahn, J. (2015). The relationship between challenging behaviours, mood and interest/pleasure in adults with severe and profound intellectual disabilities. Journal of Intellectual Disability Research, 59(11), 1033–1041. 10.1111/jir.12202 26031694

[jar12809-bib-0010] Bigby, C. , & Beadle‐Brown, J. (2018). Improving quality of life outcomes in supported accommodation for people with intellectual disability: What makes a difference? Journal of Applied Research in Intellectual Disabilities, 31(2), e182–e200. 10.1111/jar.12291 27778426

[jar12809-bib-0011] Bowring, D. L. , Totsika, V. , Hastings, R. P. , Toogood, S. , & Griffith, G. M. (2017). Challenging behaviours in adults with an intellectual disability: A total population study and exploration of risk indices. British Journal of Clinical Psychology, 56(1), 16–32. 10.1111/bjc.12118 27878840

[jar12809-bib-0012] Cervantes, P. E. , & Matson, J. L. (2015). Comorbid symptomology in adults with autism spectrum disorder and intellectual disability. Journal of Autism and Developmental Disorders, 45, 3961–3970. 10.1007/s10803-015-2553-z 26254894

[jar12809-bib-0013] Clark, M. , Crocker, A. G. , & Morin, D. (2016). Victimization history and aggressive behavior among adults with intellectual disabilities: The mediating role of mental health. International Journal of Forensic Mental Health, 15(4), 301–311. 10.1080/14999013.2016.1228087

[jar12809-bib-0014] Cooper, S. A. , Smiley, E. , Jackson, A. , Finlayson, J. , Allan, L. , Mantry, D. , & Morrison, J. (2009). Adults with intellectual disabilities: Prevalence, incidence and remission of aggressive behaviour and related factors. Journal of Intellectual Disability Research, 53(3), 217–232. 10.1111/j.1365-2788.2008.01127.x 19178617

[jar12809-bib-0015] Crocker, A. G. , Mercier, C. , Lachapelle, Y. , Brunet, A. , Morin, D. , & Roy, M. E. (2006). Prevalence and types of aggressive behaviour among adults with intellectual disabilities. Journal of Intellectual Disability Research, 50(9), 652–661. 10.1111/j.1365-2788.2006.00815.x 16901292

[jar12809-bib-0016] Crocker, A. G. , Prokić, A. , Morin, D. , & Reyes, A. (2014). Intellectual disability and co‐occurring mental health and physical disorders in aggressive behaviour. Journal of Intellectual Disability Research, 58(11), 1032–1044. 10.1111/jir.12080 23952483

[jar12809-bib-0017] Davies, B. E. , Frude, N. , Jenkins, R. , Hill, C. , & Harding, C. (2015). A study examining the relationship between alexithymia and challenging behaviour in adults with intellectual disability. Journal of Intellectual Disability Research, 59(11), 1022–1032. 10.1111/jir.12186 25683670

[jar12809-bib-0018] Didden, R. , Embregts, P. , van der Toorn, M. , & Laarhoven, N. (2009). Substance abuse, coping strategies, adaptive skills and behavioral and emotional problems in clients with mild to borderline intellectual disability admitted to a treatment facility: A pilot study. Research in Developmental Disabilities, 30, 927–932. 10.1016/j.ridd.2009.01.002 19217753

[jar12809-bib-0019] Dilworth, J. A. , Phillips, N. , & Rose, J. (2011). Factors relating to staff attributions of control over challenging behaviour. Journal of Applied Research in Intellectual Disabilities, 24(1), 29–38. 10.1111/j.1468-3148.2010.00570.x

[jar12809-bib-0020] Drieschner, K. H. , Marrozos, I. , & Regenboog, M. (2013). Prevalence and risk factors of inpatient aggression by adults with intellectual disabilities and severe challenging behaviour: A long‐term prospective study in two Dutch treatment facilities. Research in Developmental Disabilities, 34(8), 2407–2418. 10.1016/j.ridd.2013.04.008 23711630

[jar12809-bib-0021] Embregts, P. J. C. M. , Didden, R. , Huitink, C. , & Schreuder, N. (2009). Contextual variables affecting aggressive behaviour in individuals with mild to borderline intellectual disabilities who live in a residential facility. Journal of Intellectual Disability Research, 53, 255–264. 10.1111/j.1365-2788.2008.01132.x 19178616

[jar12809-bib-0022] Embregts, P. , Kroezen, M. , Mulder, E. , Van Bussel, C. , Vandernagel, J. , Budding, M. , … Wieland, J. (2019). Multidisciplinaire Richtlijn Probleemgedrag bij volwassenen met een verstandelijke beperking.

[jar12809-bib-0023] Emerson, E. (2001). Challenging behaviour: Analysis and intervention in people with severe intellectual disabilities (2nd ed.). Cambridge University Press.

[jar12809-bib-0024] Emerson, E. , Kiernan, C. , Alborz, A. , Reeves, D. , Mason, H. , Swarbrick, R. , Mason, L. , & Hatton, C. (2001). The prevalence of challenging behaviors: A total population study. Research in Developmental Disabilities, 22(1), 77–93. 10.1016/S0891-4222(00)00061-5 11263632

[jar12809-bib-0025] Esbensen, A. J. , & Benson, B. A. (2006). A prospective analysis of life events, problem behaviours and depression in adults with intellectual disability. Journal of Intellectual Disability Research, 50(4), 248–258. 10.1111/j.1365-2788.2005.00816.x 16507029

[jar12809-bib-0026] Flynn, S. , Vereenooghe, L. , Hastings, R. P. , Adams, D. , Cooper, S.‐A. , Gore, N. , Hatton, C. , Hood, K. , Jahoda, A. , Langdon, P. E. , McNamara, R. , Oliver, C. , Roy, A. , Totsika, V. , & Waite, J. (2017). Measurement tools for mental health problems and mental well‐being in people with severe or profound intellectual disabilities: A systematic review. Clinical Psychology Review, 57, 32–44. 10.1016/j.cpr.2017.08.006 28821007

[jar12809-bib-0027] Hall, W. J. (2018). Psychosocial risk and protective factors for depression among lesbian, gay, bisexual, and queer youth: A systematic review. Journal of Homosexuality, 65(3), 263–316. 10.1080/00918369.2017.1317467 28394718 PMC5634914

[jar12809-bib-0028] Hanley, G. P. , Iwata, B. A. , & McCord, B. E. (2003). Functional analysis of problem behavior: A review. Journal of Applied Behavior Analysis, 36(2), 147–185. 10.1901/jaba.2003.36-147 12858983 PMC1284431

[jar12809-bib-0029] Hartley, S. L. , & MacLean, W. E. (2007). Staff‐averse challenging behaviour in older adults with intellectual disabilities. Journal of Applied Research in Intellectual Disabilities, 20(6), 519–528. 10.1111/j.1468-3148.2006.00354.x

[jar12809-bib-0030] Hemmings, C. P. , Gravestock, S. , Pickard, M. , & Bouras, N. (2006). Psychiatric symptoms and problem behaviours in people with intellectual disabilities. Journal of Intellectual Disability Research, 50(4), 269–276. 10.1111/j.1365-2788.2006.00827.x 16507031

[jar12809-bib-0031] Hensel, J. M. , Lunsky, Y. , & Dewa, C. S. (2014). Staff perception of aggressive behaviour in community services for adults with intellectual disabilities. Community Mental Health Journal, 50(6), 743–751. 10.1007/s10597-013-9636-0 23949541

[jar12809-bib-0032] Horovitz, M. , Matson, J. L. , Hattier, M. A. , Tureck, K. , & Bamburg, J. W. (2013). Challenging behaviors in adults with intellectual disability: The effects of race and autism spectrum disorders. Journal of Mental Health Research in Intellectual Disabilities, 6(1), 1–13. 10.1080/19315864.2011.605989

[jar12809-bib-0033] Hurley, A. D. (2008). Depression in adults with intellectual disability: Symptoms and challenging behaviour. Journal of Intellectual Disability Research, 52(11), 905–916. 10.1111/j.1365-2788.2008.01113.x 18680532

[jar12809-bib-0034] Kerr, M. , Gil‐Nagel, A. , Glynn, M. , Mula, M. , Thompson, R. , & Zuberi, M. (2013). Treatment of behavioral problems in intellectually disabled adult patients with epilepsy. Epilepsia, 54, 34–40. 10.1111/epi.12103 23458464

[jar12809-bib-0035] Koritsas, S. , & Iacono, T. (2015). Predictors of challenging behaviour in adults with intellectual disability. Advances in Mental Health and Intellectual Disabilities, 9(6), 312–326. 10.1108/AMHID-06-2015-0029

[jar12809-bib-0036] Larson, F. V. , Alim, N. , & Tsakanikos, E. (2011). Attachment style and mental health in adults with intellectual disability: Self‐reports and reports by carers. Advances in Mental Health and Intellectual Disabilities, 5(3), 15–23. 10.1108/20441281111142585

[jar12809-bib-0037] LaVigna, G. W. , & Willis, T. J. (2012). The efficacy of positive behavioural support with the most challenging behaviour: The evidence and its implications. Journal of Intellectual & Developmental Disability, 37(3), 185–195. 10.3109/13668250.2012.696597 22774760

[jar12809-bib-0038] Lindsay, W. R. , Carson, D. , Holland, A. J. , Taylor, J. L. , O'Brien, G. , & Wheeler, J. R. (2013). The impact of known criminogenic factors on offenders with intellectual disability: Previous findings and new results on ADHD. Journal of Applied Research in Intellectual Disabilities, 26(1), 71–80. 10.1111/jar.12011 23255380

[jar12809-bib-0039] Lloyd, B. P. , & Kennedy, C. H. (2014). Assessment and treatment of challenging behaviour for individuals with intellectual disability: A research review. Journal of Applied Research in Intellectual Disabilities, 27(3), 187–199. 10.1111/jar.12089 24464965

[jar12809-bib-0040] Lowe, K. , Allen, D. , Jones, E. , Brophy, S. , Moore, K. , & James, W. (2007). Challenging behaviours: Prevalence and topographies. Journal of Intellectual Disability Research, 51(8), 625–636. 10.1111/j.1365-2788.2006.00948.x 17598876

[jar12809-bib-0041] Lundqvist, L. O. (2013). Prevalence and risk markers of behavior problems among adults with intellectual disabilities: A total population study in Örebro County. Sweden. Research in Developmental Disabilities, 34(4), 1346–1356. 10.1016/j.ridd.2013.01.010 23417139

[jar12809-bib-0042] Lunsky, Y. , Raina, P. , & Jones, J. (2012). Relationship between prior legal involvement and current crisis for adults with intellectual disability. Journal of Intellectual & Developmental Disability, 37(2), 163–168. 10.3109/13668250.2012.685149 22563692

[jar12809-bib-0043] Matson, J. L. , Fodstad, J. C. , & Rivet, T. T. (2009). The relationship of social skills and problem behaviors in adults with intellectual disability and autism or PDD‐NOS. Research in Autism Spectrum Disorders, 3(1), 258–268. 10.1016/j.rasd.2008.07.001

[jar12809-bib-0044] Matson, J. L. , & Rivet, T. T. (2008). The effects of severity of autism and PDD‐NOS symptoms on challenging behaviors in adults with intellectual disabilities. Journal of Developmental & Physical Disabilities, 20(1), 41–51. 10.1007/s10882-007-9078-0

[jar12809-bib-0045] National Institute for Health and Clinical Excellence (2015). Challenging behaviour and learning disabilities: Prevention and interventions for people with learning disabilities whose behaviour challenges. Retrieved from https://www.nice.org.uk/guidance/ng11/resources/challenging‐behaviour‐and‐learning‐disabilities‐prevention‐and‐interventionsfor‐people‐with‐learning‐disabilities‐whose‐behaviour‐challenges‐1837266392005 26180881

[jar12809-bib-0046] National Institutes of Health (2014). Quality assessment tool for observational cohort and cross‐sectional studies. National Heart, Lung, and Blood Institute Retrieved from www.nhlbi.nih.gov/health‐pro/guidelines/indevelop/cardiovascular‐risk‐reduction/tools/cohort

[jar12809-bib-0047] Nøttestad, J. A. , & Linaker, O. M. (2002). Predictors for attacks on people after deinstitutionalization. Journal of Intellectual Disability Research, 46(6), 493–502. 10.1046/j.1365-2788.2002.00418.x 12354320

[jar12809-bib-0048] Novaco, R. W. , & Taylor, J. L. (2004). Assessment of anger and aggression in male offenders with developmental disabilities. Psychological Assessment, 16, 42–50. 10.1037/1040-3590.16.1.42 15023091

[jar12809-bib-0049] Owen, D. M. , Hastings, R. P. , Noone, S. J. , Chinn, J. , Harman, K. , Roberts, J. , & Taylor, K. (2004). Life events as correlates of problem behavior and mental health in a residential population of adults with developmental disabilities. Research in Developmental Disabilities, 25, 309–320. 10.1016/j.ridd.2004.01.003 15193667

[jar12809-bib-0050] Peña‐Salazar, C. , Arrufat, F. , Santos, J. M. , Fontanet, A. , González‐Castro, G. , Más, S. , Roura‐Poch, P. , & Valdés‐Stauber, J. (2018). Underdiagnosis of psychiatric disorders in people with intellectual disabilities: Differences between psychiatric disorders and challenging behaviour. Journal of Intellectual Disabilities, 24(3), 1744629518798259 10.1177/1744629518798259 30185101

[jar12809-bib-0051] Phillips, N. , & Rose, J. (2010). Predicting placement breakdown: Individual and environmental factors associated with the success or failure of community residential placements for adults with intellectual disabilities. Journal of Applied Research in Intellectual Disabilities, 23(3), 201–213. 10.1111/j.1468-3148.2009.00530.x

[jar12809-bib-0052] Rojahn, J. , Matson, J. L. , Naglieri, J. A. , & Mayville, E. (2004). Relationships between psychiatric conditions and behavior problems among adults with mental retardation. American Journal on Mental Retardation, 109(1), 21–33. 10.1352/0895-8017(2004)109<21:RBPCAB>2.0.CO;2 14651452

[jar12809-bib-0053] Rojahn, J. , Wilkins, J. , Matson, J. L. , & Boisjoli, J. (2010). A comparison of adults with intellectual disabilities with and without ASD on parallel measures of challenging behaviour: The Behavior Problems Inventory‐01 (BPI‐01) and autism spectrum disorders‐behavior problems for intellectually disabled adults (ASD‐BPA). Journal of Applied Research in Intellectual Disabilities, 23, 179–185. 10.1111/j.1468-3148.2009.00519.x

[jar12809-bib-0054] Rose, J. (2011). How do staff psychological factors influence outcomes for people with developmental and intellectual disability in residential services? Current Opinion in Psychiatry, 24(5), 403–407. 10.1097/YCO.0b013e3283476b0b 21587078

[jar12809-bib-0055] Ross, E. , & Oliver, C. (2002). The relationship between levels of mood, interest and pleasure and ‘challenging behaviour’ in adults with severe and profound intellectual disability. Journal of Intellectual Disability Research, 46(3), 191–197. 10.1046/j.1365-2788.2002.00397.x 11896804

[jar12809-bib-0056] Royal College of Psychiatrists, British Psychological Society and Royal College of Speech and Language Therapists (2007). Challenging behaviour: A unified approach. Clinical and service guidelines for supporting people with learning disabilities who are at risk of receiving abusive or restrictive practices. Royal College of Psychiatrists London.

[jar12809-bib-0057] Sappok, T. , Budczies, J. , Dziobek, I. , Bölte, S. , Dosen, A. , & Diefenbacher, A. (2014). The missing link: Delayed emotional development predicts challenging behavior in adults with intellectual disability. Journal of Autism and Developmental Disorders, 44, 786–800. 10.1007/s10803-013-1933-5 24002416

[jar12809-bib-0058] Schalock, R. L. , Borthwick‐Duffy, S. A. , Bradley, V. J. , Buntinx, W. H. , Coulter, D. L. , Craig, E. M. , Gomez, S. C. , Luckasson, R. , Reeve, A. , Shogren, K. A. , Snell, M. E. , Spreat, S. , Tasse, M. J. , Thompson, J. R. , Verdugo‐Alonso, M. A. , Wehmeyer, M. L. , & Yeager, M. H. (2010). Intellectual disability: Definition, classification, and systems of supports (11th ed.). American Association on Intellectual and Developmental Disabilities.

[jar12809-bib-0059] Sorgi, P. , Ratey, J. J. , Knoedler, D. W. , Markert, R. J. , & Reichman, M. (1991). Rating aggression in the clinical setting: A retrospective adaptation of the Overt Aggression Scale: Preliminary results. The Journal of Neuropsychiatry and Clinical Neurosciences, 3, S52–S56. 1687961

[jar12809-bib-0060] Tenneij, N. H. , Didden, R. , Stolker, J. J. , & Koot, H. M. (2009). Markers for aggression in inpatient treatment facilities for adults with mild to borderline intellectual disability. Research in Developmental Disabilities, 30(6), 1248–1257. 10.1016/j.ridd.2009.04.006 19464143

[jar12809-bib-0061] Thakker, Y. , Bamidele, K. , Ali, A. , & Hassiotis, A. (2012). Mental health and challenging behaviour: An overview of research and practice. Advances in Mental Health and Intellectual Disabilities, 6(5), 249–258. 10.1108/20441281211261131

[jar12809-bib-0062] Thorson, R. , Matson, J. , Rojahn, J. , & Dixon, D. (2008). Behaviour problems in institutionalised people with intellectual disability and schizophrenia spectrum disorders. Journal of Intellectual & Developmental Disability, 33(4), 316–322. 10.1080/13668250802441649 19039691

[jar12809-bib-0063] Totsika, V. , Toogood, S. , Hastings, R. P. , & Lewis, S. (2008). Persistence of challenging behaviours in adults with intellectual disability over a period of 11 years. Journal of Intellectual Disability Research, 52(5), 446–457. 10.1111/j.1365-2788.2008.01046.x 18331560

[jar12809-bib-0064] Tsiouris, J. A. , Kim, S. Y. , Brown, W. T. , & Cohen, I. L. (2011). Association of aggressive behaviours with psychiatric disorders, age, sex and degree of intellectual disability: A large‐scale survey. Journal of Intellectual Disability Research, 55, 636–649. 10.1111/j.1365-2788.2011.01418.x 21492292

[jar12809-bib-0065] Turygin, N. , Matson, J. , MacMillan, K. , & Konst, M. (2013). The relationship between challenging behavior and symptoms of depression in intellectually disabled adults with and without autism spectrum disorders. Journal of Developmental & Physical Disabilities, 25(4), 475–484. 10.1007/s10882-012-9321-1

[jar12809-bib-0066] Tyrer, F. , McGrother, C. W. , Thorp, C. F. , Donaldson, M. , Bhaumik, S. , Watson, J. M. , & Hollin, C. (2006). Physical aggression towards others in adults with learning disabilities: Prevalence and associated factors. [Erratum appears in J Intellect Disabil Res. 2006 May; 50(Pt 5):395]. Journal of Intellectual Disability Research, 50(Pt 4), 295–304. 16507034 10.1111/j.1365-2788.2005.00774.x

[jar12809-bib-0067] Willems, A. P. A. M. , Embregts, P. J. C. M. , Bosman, A. M. T. , & Hendriks, A. H. C. (2014). The analysis of challenging reactions: Influences on interactive behaviour of staff towards clients with intellectual disabilities. Journal of Intellectual Disability Research, 58, 1072–1082. 23480642 10.1111/jir.12027

